# HIV-1 Infection and First Line ART Induced Differential Responses in Mitochondria from Blood Lymphocytes and Monocytes: The ANRS EP45 “Aging” Study

**DOI:** 10.1371/journal.pone.0041129

**Published:** 2012-07-19

**Authors:** Sophie Perrin, Jonathan Cremer, Patrice Roll, Olivia Faucher, Amélie Ménard, Jacques Reynes, Pierre Dellamonica, Alissa Naqvi, Joëlle Micallef, Elisabeth Jouve, Catherine Tamalet, Caroline Solas, Christel Pissier, Isabelle Arnoux, Corine Nicolino-Brunet, Léon Espinosa, Nicolas Lévy, Elise Kaspi, Andrée Robaglia-Schlupp, Isabelle Poizot-Martin, Pierre Cau

**Affiliations:** 1 Inserm UMR 910, Aix-Marseille Univ, Marseille, France; 2 Laboratoire de Biologie Cellulaire, CHU (Centre Hospitalier Universitaire) La Timone AP-HM (Assistance Publique - Hôpitaux de Marseille), Marseille, France; 3 Service d’Immuno-Hématologie Clinique, CHU (Centre Hospitalier Universitaire) Sainte Marguerite AP-HM (Assistance Publique - Hôpitaux de Marseille), Marseille, France; 4 Département des Maladies Infectieuses et Tropicales, CHRU (Centre Hospitalier Régional et Universitaire) Gui-de-Chauliac, Montpellier, France; 5 Service d’Infectiologie, CHU (Centre Hospitalier Universitaire) L’Archet 1, Nice, France; 6 Centre d’Investigation Clinique - Unité de Pharmacologie Clinique et d’Evaluations Thérapeutiques (CIC-UPCET), CHU (Centre Hospitalier Universitaire) La Timone AP-HM (Assistance Publique - Hôpitaux de Marseille), Marseille, France; 7 Fédération de Microbiologie Clinique, CHU (Centre Hospitalier Universitaire) La Timone AP-HM (Assistance Publique - Hôpitaux de Marseille), Marseille, France; 8 URMITE CNRS-IRD UMR 6236, Aix-Marseille Univ, Marseille, France; 9 Laboratoire de Pharmacocinétique et de Toxicologie, CHU (Centre Hospitalier Universitaire) La Timone AP-HM (Assistance Publique - Hôpitaux de Marseille), Marseille, France; 10 Inserm UMR U911, Aix-Marseille Univ, Marseille, France; 11 Laboratoire d’Hématologie, CHU (Centre Hospitalier Universitaire) La Timone AP-HM (Assistance Publique - Hôpitaux de Marseille), Marseille, France; 12 Laboratoire d’Hématologie, CHU (Centre Hospitalier Universitaire) La Conception AP-HM (Assistance Publique - Hôpitaux de Marseille), Marseille, France; 13 Laboratoire de Génetique Moléculaire, CHU (Centre Hospitalier Universitaire) La Timone AP-HM (Assistance Publique - Hôpitaux de Marseille), Marseille, France; University of Medicine and Dentistry of New Jersey - New Jersey Medical School, United States of America

## Abstract

**Background:**

The ANRS EP45 “Aging” study investigates the cellular mechanisms involved in the accelerated aging of HIV-1 infected and treated patients. The data reported focus on mitochondria, organelles known to be involved in cell senescence.

**Methods:**

49 HIV-1 infected patients untreated with antiretroviral therapy, together with 49 seronegative age- and sex-matched control subjects and 81 HIV-1 infected and treated patients, were recruited by 3 AIDS centres (Marseille, Montpellier, Nice; France; http://clinicaltrials.gov/, NCT01038999). In more than 88% of treated patients, the viral load was <40 copies/ml and the CD4+ cell count was >500/mm^3^. ROS (reactive oxygen species) production and ΔΨm (inner membrane potential) were measured by flow cytometry in blood lymphocytes and monocytes (functional parameters). Three mitochondrial network quantitative morphological parameters were computed using confocal microscopy and image analysis. Three PBMC mitochondrial proteins (porin and subunits 2 and 4 of cytochrome C oxidase encoded by mtDNA or nuclear DNA, respectively) were analysed by western blotting.

**Results:**

Quantitative changes in PBMC mitochondrial proteins were not induced by either HIV-1 infection or ART. Discriminant analysis integrating functional (ROS production and ΔΨm) or morphological (network volume density, fragmentation and branching) parameters revealed HIV-1 infection and ART differential effects according to cell type. First line ART tended to rescue lymphocyte mitochondrial parameters altered by viral infection, but induced slight changes in monocytes. No statistical difference was found between the effects of three ART regimens on mitochondrial parameters. Correlations between functional parameters and viral load confirmed the damaging effects of HIV-1 in lymphocyte mitochondria.

**Conclusions:**

In patients considered to be clinically stable, mitochondria exhibited functional and morphological modifications in PBMCs resulting from either direct or indirect effects of HIV-1 infection (lymphocytes), or from first line ART (monocytes). Together with other tissue impairments, these changes may contribute to global aging.

**Trial Registration:**

ClinicalTrials.gov NCT01038999 NCT01038999

## Introduction

Since 1996, antiretroviral therapy (ART) has increased life expectancy in HIV-infected patients who exhibit aging-related diseases [1]. The ANRS EP45 “Aging” study investigates the cellular mechanisms in peripheral blood mononuclear cells (PBMCs) that lead to aging in treated or untreated (naive) HIV-1 infected patients.

This paper is focused on mitochondria, the main energy-producing factories in the cell, which are known to be involved in ROS production [2], and also in antiviral innate immune defense [3] and aging [4]. Moreover, direct targeting of mitochondria either by proteins [5] and miRNAs [6] encoded by HIV, or by ART [7], [8], is thought to trigger apoptosis [9].

The production of ATP by the respiratory chain involves multiheteromeric enzymatic complexes located in the inner mitochondrial membrane (IMM). Protons are pumped from the mitochondrial matrix to the intermembrane space to establish an electrochemical gradient that results in the IMM potential (ΔΨm) required for ATP synthesis. More than 90% of the oxygen in tissues is consumed by mitochondria, and between 1 and 2–5% of the oxygen is transformed into reactive oxygen species (ROS) as respiratory chain by-products [10]. At low concentrations, ROS can function as signaling molecules [11]. However, at high concentrations, ROS may cause damage to cellular components even though the cell possesses sophisticated antioxidant defense systems [12]. Overproduction of ROS may therefore directly decrease ΔΨm and lead to a lowered ATP supply, and may also cause mitochondrial network fragmentation and subsequent mitochondrial autophagy (mitophagy), cell apoptosis or cell senescence [13].

Mitochondrial network dynamics, cell apoptosis and autophagy exhibit close reciprocal relationships with innate antiviral signaling and mitochondrial morphological or functional (ROS production, ΔΨm and ATP supply) parameters. These events are coordinated by common mitochondrial or cytosolic partner proteins that are regulated by post-translational modifications [14].

Mitochondria form a dynamic reticulum that is continuously remodeled by balanced fission and fusion (or “kiss and run”) events controlled by two sets of outer (OMM) and inner (IMM) mitochondrial membrane specific proteins [15]. Fission events often generate uneven daughter mitochondria, with the fusion-competent mitochondria exhibiting a higher ΔΨm. Fusion-incompetent mitochondria are characterized by a low ΔΨm due to the accumulation of ROS-damaged molecules and mutated mtDNA [16], and are targeted for degradation by mitophagy [17].

Molecular partners that link ROS overproduction, ΔΨm decrease, mitochondrial fission and mitophagy through the sequential recruitment and interaction of cytosolic proteins, OMM GTPases, IMM GTPases and oxidative phosphorylation (OXPHOS) complexes, have been implicated in the pathogenesis of Parkinson’s disease [18].

ROS [3] and ΔΨm [19] also regulate the innate immune response triggered by cytosolic RNA helicases of the RLR (RIG-1-like receptors) family, via activation of the MAVS (Mitochondrial Antiviral Signaling) protein [20]. HIV escapes from antiviral signaling and innate immune responses through RIG-1 lysosomal degradation induced by HIV protease [21].

HIV-encoded proteins or miRNAs trigger mitochondrial-mediated apoptosis, which may explain the progressive decline in CD4+ T cells in infected patients [9], [22]. Apoptosis has been shown to be triggered by ROS overproduction [23], ΔΨm lowering [24] or network disruption [25].

ART mainly targets two steps of the HIV lifecycle. Nucleoside reverse transcriptase inhibitors (NRTIs) and non-nucleoside reverse transcriptase inhibitors (NNRTIs) block reverse transcription, whereas ritonavir-boosted protease inhibitors (PI/r) prevent the cleavage of HIV-encoded gag-pol proteins. A combination of molecules from these two groups currently represents the most common HIV infection treatment. However, ART with primarily first generation NRTIs can cause mitochondrial toxicity through mitochondrial DNA polymerase γ inhibition [26], which may contribute to patient aging [27]. Only limited data are available regarding toxicity due to NNRTIs and PIs [7].

We used PBMCs as an easily available cell type targeted by both HIV-1 and ART. As a direct or indirect response of HIV-1 infection, flow cytometry demonstrated that ROS production and mitochondrial ΔΨm were altered in lymphocytes but not in monocytes from ART naive patients. ART partially rescued these lymphocyte parameters but induced slight mitochondrial changes in monocytes. Mitochondrial network morphology parameters (volume, fragmentation and branching) observed by confocal microscopy in these two cell types confirmed the functional mitochondrial results. Network fragmentation was stable regardless of subject status, and an increase in network volume and branching argued against mitophagy or mitochondria-linked apoptosis related to ROS and ΔΨm changes. No modifications in the amounts of mitochondrial proteins were observed in PBMCs. Even in patients considered to be clinically stable, the changes in PBMCs reported here, together with alteration of mitochondrial functional and morphological parameters in other tissues, may contribute to aging in HIV-1 infected patients.

## Methods

### ANRS EP45 Study & Participant Characteristics

The ANRS EP45 “Aging” study is a cross-sectional and longitudinal (3 years) multicentre study. Patients were recruited by three specialised AIDS centres in France at Marseilles (the main coordination centre), Nice and Montpellier, and control subjects were recruited by CIC-UPCET (Pharmacologie Clinique & Evaluations Thérapeutiques, Timone Hospital) in Marseilles.

Forty-nine HIV-1 patients infected for at least 24 months but who had not received ART (ART naive), and eighty-one HIV-1 infected patients in the first line of ART for at least 12 months, were enrolled. According to their ART combination, patients were distributed into three subgroups: patients treated with 2NRTIs and PI/r; patients treated with 2NRTIs and 1NNRTI; and patients treated with 3NRTIs. Forty-nine seronegative control subjects were age- and sex-matched with the ART naive patients.

The basic demographic, clinical and biological parameters of the study participants, together with their ART combinations, are detailed in Tables S1 and S2. A complete clinical and biological description of the cohort will be detailed in another report (Faucher *et al.*, in preparation). All experiments described below were performed blinded with respect to subject status (naive or treated HIV patients, control subjects). We report here the mitochondrial data measured at baseline.

### Ethics Statement

The protocol was approved by the French Health Products Safety Agency Regulatory Authority (AFSSAPS, Agence Française de Sécurité Sanitaire des Produits de Santé) and Marseille’s Ethical Committee (Comité de Protection des Personnes Sud Méditerranée I). The study was registered on the ClinicalTrials.gov web site (Identifier: NCT01038999, see supporting Protocol S1) and performed in accordance with the Declaration of Helsinki. All subjects provided written informed consent before participation.

### PBMC Isolation

The processing of blood samples is described in detail in the Methods S1. Briefly, PBMCs were isolated by Ficoll® gradient centrifugation (UNI-SEP MAXI+, Novamed) according to the manufacturer’s instructions. Leucocyte formulae were evaluated by May-Grünwald-Giemsa staining of cytospin samples. Cell viability was >97% (Methods S1, Figure S1).

### ΔΨm Measurement

ΔΨm was determined using the red/green fluorescence intensity ratio of 5,5′,6,6′-tetrachloro-1,1′,3,3′-tetraethylbenzimidazol-carbocyanine iodide probe (JC-1, Life Technologies). PBMCs were stained with 0.5 µM probe for 15 minutes at 37°C, 5% CO_2_. Pre-incubation with 10 µM carbonyl cyanide 3-chlorophenylhydrazone (CCCP, Sigma) for 20 minutes was used as a positive control for mitochondrial membrane depolarization. Cells incubated without JC-1 probe were used as auto-fluorescence negative control cells. Monocytes were labelled using CD14-PC5 antibody (Beckman Coulter Inc) according to the manufacturer’s instructions. Residual red blood cells, if any, were hypotonically lysed using a solution of 100 µM EDTA, 150 mM NH_4_Cl, 1 mM KHCO_3_, pH 7.4. HIV-1 was inactivated by fixation for 30 minutes in ice-cooled 0.5% paraformaldehyde solution. As previously described [28], we confirmed that fixation induced no changes in the fluorescence pattern. Red and green mean fluorescence intensity and standard deviation (MFI ± SD) were measured by flow cytometry (FC500, Beckman Coulter Inc) on an average of 3,000 CD14+ monocytes and 15,000 lymphocytes identified according to forward/side scatter (FSC/SSC). No CD14+ cells were observed in the lymphocyte population after gating. All data were analysed using FlowJo® software (Tree Star Inc).

### ROS Production

ROS production was detected using 5-(and-6)-chloromethyl-2′,7′-dichlorodihydro-fluorescein diacetate acetyl ester probe (CM-H_2_DCFDA, Life Technologies). PBMCs were incubated with 5 µM probe for 30 minutes at 37°C, 5% CO_2_. Co-incubation of CM-H_2_DCFDA with 50 ng/ml of phorbol 12-myristate 13-acetate (PMA, LC Laboratories) was used as a positive control. Cells incubated without CM-H_2_DCFDA probe were used as auto-fluorescence negative control cells. Staining of CD14-monocytes, lysis of residual red blood cells (if any), fixation and flow cytometry analysis were performed as described above.

### Analysis of the Mitochondrial Network

The mitochondrial network was stained using Mitotracker® Red CMXRos probe (Life Technologies). PBMCs were stained with 25 nM probe for 15 minutes at 37°C, 5% CO_2_. After fixation for 15 minutes at room temperature (RT) with 4% paraformaldehyde solution, cytospin samples of PBMCs were prepared at 23 g for 5 minutes and nuclei were labelled with DAPI (0.1 µg/ml, Sigma) for 10 minutes at RT. Slides were mounted using FluorSave™ reagent (Merck). A single confocal slide (Leica SP5, LAS 6000 AF Leica software, Leica Microsystems) was recorded at 100×magnification from 30 lymphocytes and 30 monocytes identified according to their nuclear size and shape after DAPI staining. Three individuals from each of the 5 groups (uninfected control, ART naive, 3 ART combinations) were selected for analysis. The individuals were chosen on the basis of possessing maximal (44 to 71), minimal (15 to 22) and medium (31 to 37) ROS values in high-ROS producing lymphocytes from flow cytometry data. ΔΨm was confirmed as being inversely correlated with ROS values in the total lymphocyte population (Figure S2). Image analysis was performed using Image J 1.44 d software (http://rsbweb.nih.gov/ij/index.html). Three mitochondrial network stereological parameters (volume density, fragmentation and branching) were calculated for lymphocytes and monocytes using a specific macro designed by Léon Espinosa (available on request). Figure S3 shows the macro steps and the corresponding results for 4 different monocytes.

### Western Blotting of Mitochondrial Proteins

Total PBMC proteins from 160 cohort participants were extracted and analysed by a standard western blotting procedure, as detailed in Methods S1.

### Statistical Analysis

Statistical analysis was conducted using SAS software, version 9.2 (SAS Institute Inc). ΔΨm, ROS, mitochondrial network and quantitative protein parameters were compared between groups by analysis of variance or Kruskall-Wallis test. Pairwise comparisons were conducted using the Bonferroni method for multiple comparisons. Relationships between parameters were assessed by Pearson’s correlation coefficient. XLSTAT® software (AddinSoft) was used for box plot drawings and discriminant analysis, whereas comparison between groups used Wilks’ Lambda (Rao’s approximation) or Roy’s greatest root tests [29]. Percentage changes were calculated using mean values. p values <0.05 were considered as statistically significant.

## Results

### Identification of Subpopulations of Lymphocytes and Monocytes by Flow Cytometric Measurements of Two Mitochondrial Functional Parameters: ROS Production and Inner Membrane Potential ΔΨm

**Figure 1:**
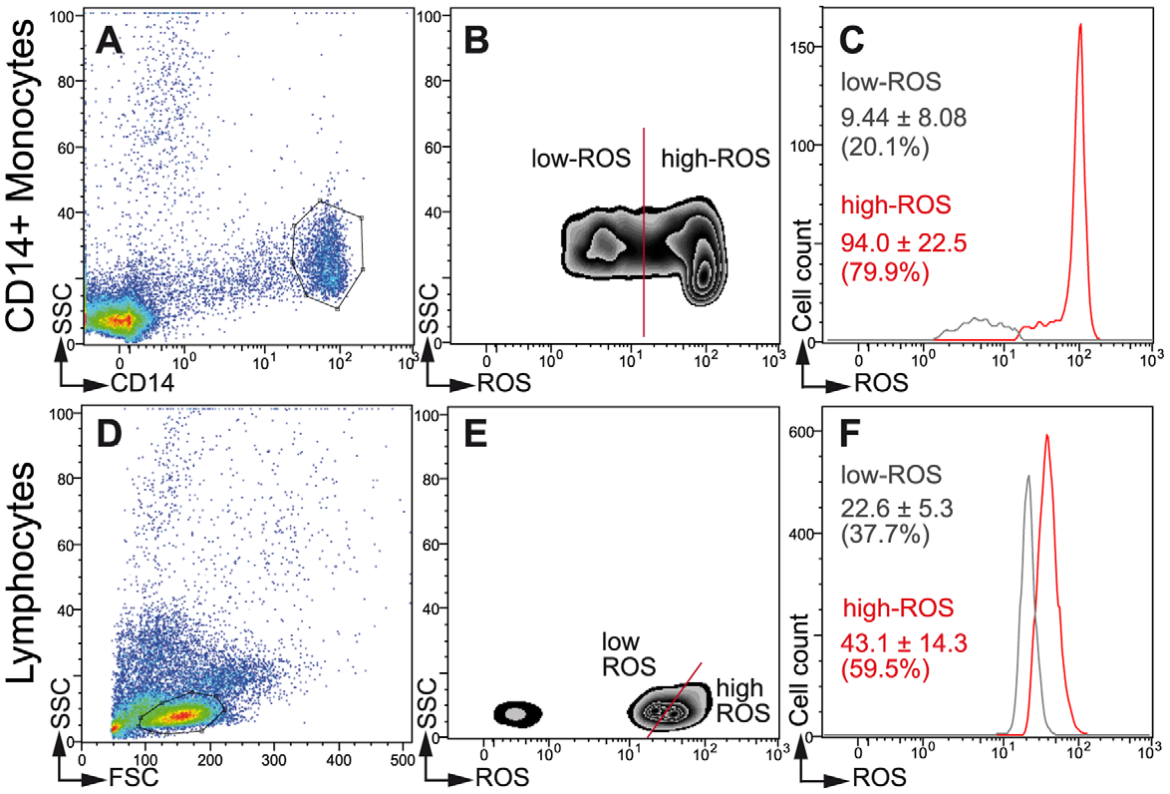
Flow cytometric analysis of ROS production revealed two subpopulations of both CD14+ monocytes and lymphocytes. (**A to C**) Monocytes. (**A**) CD14+/SSC monocyte gating. (**B**) Representative zebra plot based on side scatter and ROS production showing low- and high-ROS producing cell subpopulations. (**C**) Histogram illustrating ROS production as a function of cell count (low-ROS production, gray; high-ROS production, red). ROS production levels (MFI ± SD) are indicated as percentages relative to CD14+ monocytes. The percentages of high-ROS monocytes were not statistically different between the groups: Control: 75.8±21.7%; ART naive: 70.7±19.4%; 2NRTI+1PI/r: 71.1±21.1%; 2NRTI+1NNRTI: 71.0±22.8%; 3NRTI: 66.2±23.7%. (**D to F**) Lymphocytes. (**D**) FSC/SSC lymphocyte gating. (**E**) Representative zebra plot based on side scatter and ROS production showing low- and high-ROS producing cell subpopulations. (**F**) Histogram illustrating ROS production as a function of cell count (low-ROS production, gray; high-ROS production, red). ROS production levels (MFI ± SD) are indicated as percentages relative to FSC/SSC-selected lymphocytes. The percentages of high-ROS lymphocytes were not statistically different between the groups: Control: 55.4±12.7%; ART naive: 48.0±14.1%; 2NRTI+1PI/r: 55.1±13.8%; 2NRTI+1NNRTI: 50.1±14.1%; 3NRTI: 47.6±15.0%. ROS production by the low- and high-ROS subpopulations was statistically different in both HIV and ART patients.

Evidence for high- and low-ROS producing cells in all patients (monocytes), or in 72–77% of patients according to HIV-1 and ART status (lymphocytes) is shown in representative zebra plots and histograms of CD14+ monocytes (Figures 1B, 1C) or lymphocytes (Figures 1E, 1F). High-ROS cells significantly produced (p<0.0001) 8 to 10 times (monocytes) or 1.5 to 2 times (lymphocytes) more ROS than low-ROS cells. Among the five participant groups, no significant differences in the percentage of high-ROS producing monocytes (from 66 to 76%) or lymphocytes (from 48 to 55%) were observed. Further characterisation of these two lymphocyte subpopulations has been achieved in two small groups of control subjects, age- and sex-matched with HIV-1 infected and treated patients (CD4+ cell count >500/mm^3^; viral load <40 copies/ml), out of the ANRS EP45 cohort. Regardless of HIV-1 status, four lymphocytes subtypes, CD3+, CD3+ CD4+, CD3+ CD8+ and CD3− CD19+, exhibited similar patterns of ROS production within the total lymphocyte population. This indicated that these cells were subdivided into both high- and low-ROS cells, whereas CD3− CD19− CD16+ CD56+ NK cells were always low-ROS producing cells (Figure S4). HIV-1 infected CD4+ lymphocytes presented the same pattern as CD8+ and CD19+ uninfected cells.

**Figure 2:**
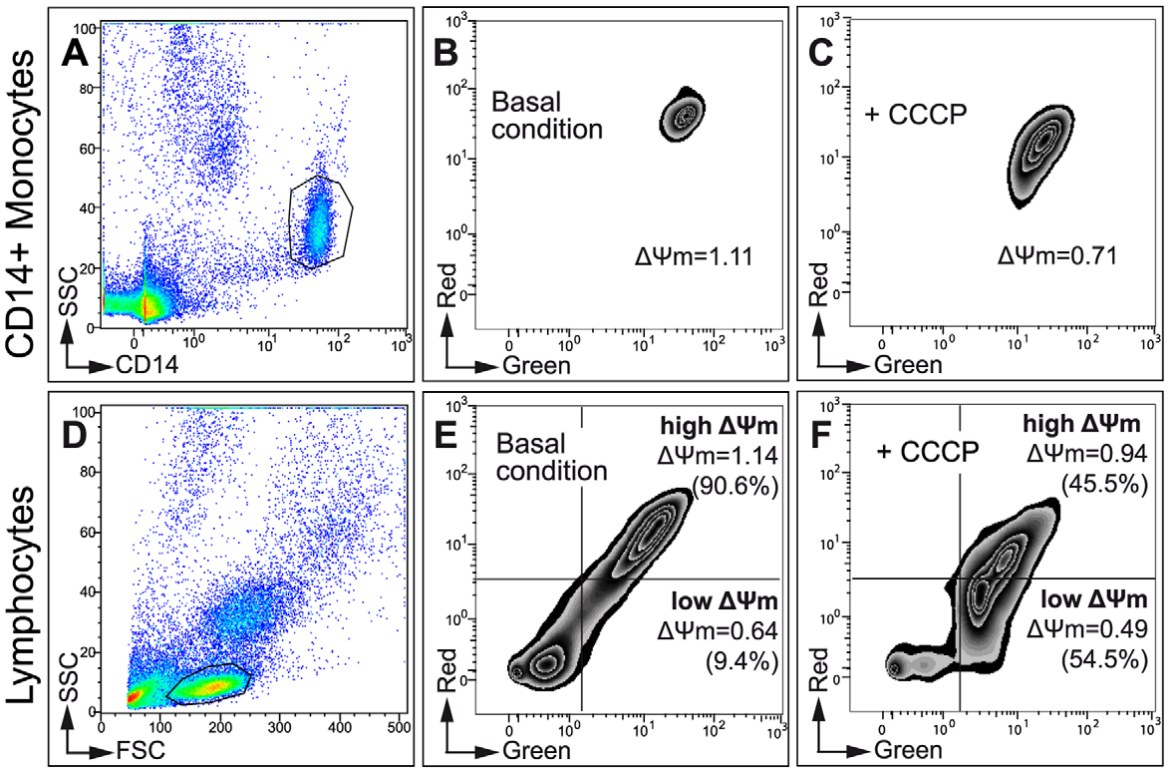
Determination of mitochondrial inner membrane potential (ΔΨm) in lymphocytes and monocytes. JC-1 is a cationic carbocyanine probe that exibits a potential-dependent accumulation in mitochondria as either a monomer at low concentrations (green fluorescence) or as aggregates at higher concentrations (red fluorescence) [28]. Therefore, the red/green fluorescence intensity ratio illustrates ΔΨm. (**A to C**) Monocytes. (**A**) CD14+/SSC monocyte gating. (**B**) Representative zebra plot based on side scatter and ΔΨm. (**C**) Representative zebra plot based on side scatter and ΔΨm after inhibition of ΔΨm with CCCP (control experiment). (**D to F**) Lymphocytes. (**D**) FSC/SSC lymphocyte gating. (**E**) Representative zebra plot based on side scatter and ΔΨm. Two lymphocyte subpopulations were distinguished (low-ΔΨm, bottom right quadrants; high-ΔΨm, top right quadrants). The top and bottom right quadrants indicate the ΔΨm mean values (cell percentages). The percentages of low-ΔΨm lymphocytes were: Control: 14.7±8.2%; ART naive: 12.56±4.4% (statistically different from control); 2NRTI+1PI/r: 17.0±8.4%; 2NRTI+1NNRTI: 15.3±7.3%; 3NRTI: 16.3±5.1%. (**F**) Representative zebra plot based on side scatter and ΔΨm after inhibition of ΔΨm with CCCP (control experiment).

Measurements of ΔΨm by flow cytometry after incubation with the ΔΨm inhibitor, CCCP, clearly identified two subpopulations of lymphocytes (Figures 2F). These two subpopulations were separated by quadrant markers and were considered as high-ΔΨm lymphocytes with functional mitochondria (top right quadrant), and low-ΔΨm lymphocytes containing weakly functional mitochondria (bottom right quadrant). CCCP markedly decreased ΔΨm in both lymphocyte subpopulations and considerably increased the percentage of cells with weakly functional mitochondria. The same quadrant markers were then applied to lymphocytes under basal conditions. In contrast, low- and high-ΔΨm monocytes were not observed (Figures 2B, 2C).

Because the emission spectra of the probes overlapped, simultaneous measurement of ROS and ΔΨm was not performed in lymphocytes or monocytes. Therefore, the relationships, if any, between high- or low-ROS and ΔΨm remain unknown. A negative correlation (p<0.001) was highlighted between ΔΨm and ROS production within the total lymphocyte population (Figure S2), but not by monocytes (not shown).

### Multiparametric Discriminant Analysis of 8 (Lymphocytes) or 4 (Monocytes) Mitochondrial Functional Parameters Revealed Differential Responses by the Two Cell Types to Either HIV-1 Infection or ART

Because changes in low-ROS cells between infected and uninfected subjects were not observed, we focused our investigations on high-ROS cells.

**Figure 3:**
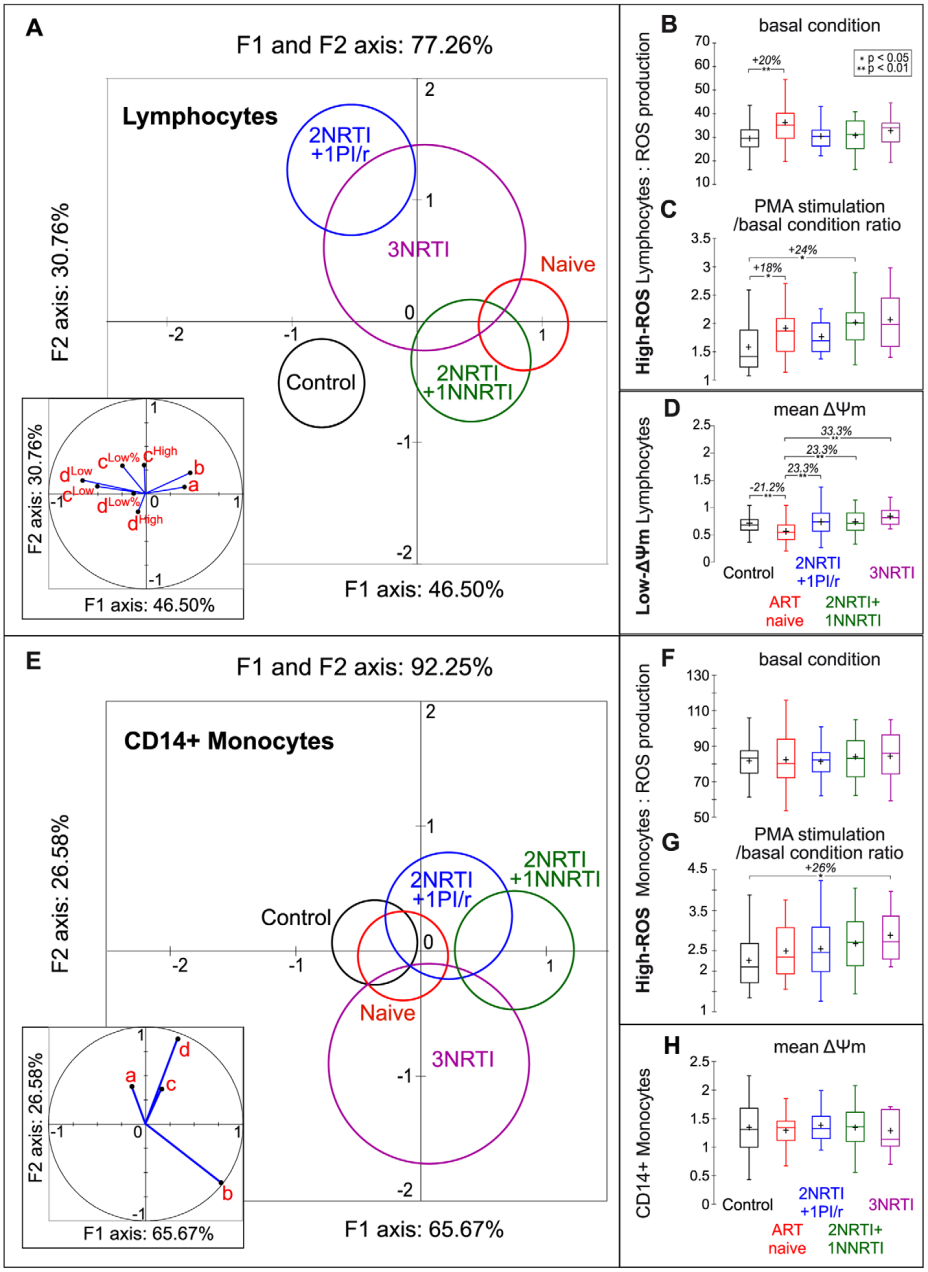
Mitochondrial functional parameters: differential effects of HIV-1 infection and ART on lymphocytes and monocytes. (**A, E**) Discriminant analysis using ROS and ΔΨm parameters. The five cohort populations are delineated by their 95% confidence circles around the means. The perpendicular axes describe the combined variance of the parameters analysed (lymphocytes: 77.26%, monocytes: 92.25%). The contributions of each parameter to the variances on the X and Y axes are shown in the insets. (**A**) Lymphocytes. ART naive patients are statistically different to control subjects. ART partially rescues mitochondrial parameter changes induced by HIV infection. Inset: The 8 mitochondrial parameters used were: a: Basal ROS production by high-ROS lymphocytes (see Figure 3B). b: PMA/Basal ratio of ROS production by high-ROS lymphocytes (see Figure 3C). c^High^: ΔΨm of high-ΔΨm lymphocytes. c^Low^: ΔΨm of low-ΔΨm lymphocytes (see Figure 3D). c^Low%^: Percentage of low-ΔΨm lymphocytes. d^High^: Basal to CCCP ΔΨm ratio of high-ΔΨm lymphocytes. d^Low^: Basal to CCCP ΔΨm ratio of low-ΔΨm lymphocytes. d^Low%^: CCCP to Basal ratio of low-ΔΨm lymphocyte percentage. (**E**) Monocytes. ART naive patients are close to control subjects whereas ART combinations are more dispersed. Inset: The 4 mitochondrial parameters used were: a: Basal ROS production by high-ROS monocytes (see Figure 3F). b: PMA to Basal ratio of ROS production by high-ROS monocytes (see Figure 3G). c: ΔΨm of monocytes (see Figure 3H). d: Basal to CCCP ΔΨm ratio of monocytes. (**B to D; F to H**) Box plots (XLSTAT) displaying 1st quartile (Q1), median, mean (displayed by ‘+’), 3rd quartile (Q3) together with both lower and upper limits were calculated as follows: lower limit = X_i_ such that {X_i_ – [Q1–1.5 (Q3– Q1)]} is the minimum and X_i_ ≥ Q1–1.5 (Q3– Q1); upper limit = Y_i_ such that { Y_i_ – [Q3+1.5 (Q3– Q1)]} is the minimum and Y_i_ ≤ Q3+1.5 (Q3– Q1). *p<0,05, **p<0,01.

Lymphocyte mitochondria ROS- and ΔΨm-related parameters in ART naive patients were statistically different to those in control subjects (p<0.0001, Figure 3A). Mitochondrial changes were partially rescued by certain ART combinations. The 2NRTI+1NNRTI population was not statistically different to ART naive patients (p = 0.181) or control subjects (p = 0.068). In contrast, 2NRTI+1PI/r patients were statistically different to both the ART naive patients (p = 0.0001) and control subjects (p = 0.006). The 3NRTI population, which exhibited the most heterogeneous pattern as shown by their large 95% confidence circle, was statistically different to the ART naive patients (p = 0.005) but not the control subjects (p = 0.081).

Among the 8 lymphocyte mitochondrial functional parameters, the changes mainly concerned basal ROS production by high-ROS cells (Figure 3B), the PMA-stimulated to basal ratio (Figure 3C), ΔΨm of low-ΔΨm lymphocytes (Figure 3D), and, to a lesser extent, the percentage of low-ΔΨm cells (not shown). Neither HIV-1 infection nor ART induced statistical differences in ΔΨm values from High-ΔΨm lymphocytes compared to control subjects (data not shown).

In contrast to lymphocytes, the 4 monocyte ROS- and ΔΨm-related parameters were unchanged in the ART naive patients compared to control subjects (p = 0.838, Figure 3E). The three ART combinations showed slight but not significant differences to the ART naive or control populations. However, a statistical difference (p = 0.023) was recorded between control subjects and 2NRTI+1NNRTI patients.

Among the 4 monocyte mitochondrial functional parameters (Figure 3F to H), changes were observed regarding the ratio of PMA-stimulated to basal ROS production by high-ROS cells (Figure 3G).

### Two Mitochondrial Functional Parameters in Lymphocytes, but Only One in Monocytes, were Correlated with Viral Load

**Figure 4:**
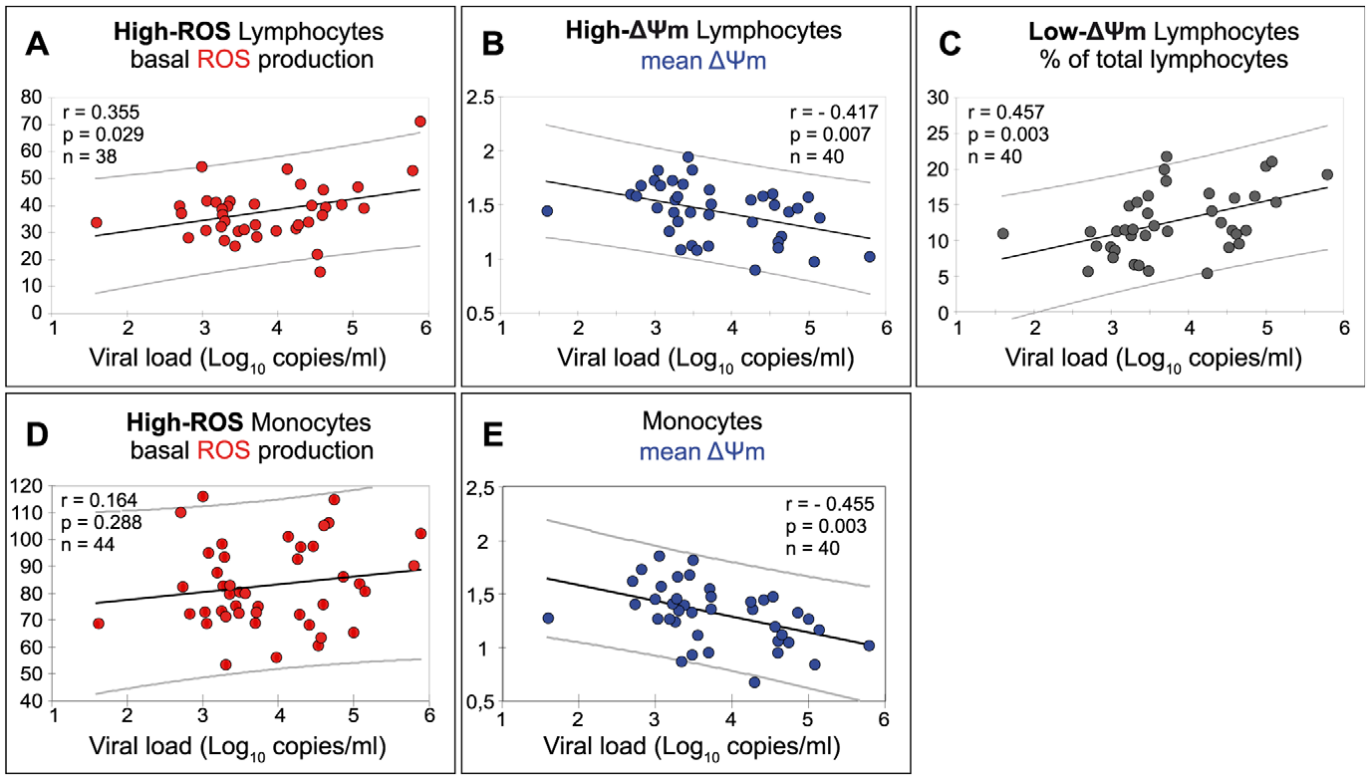
Correlation between mitochondrial functional parameters and viral load in cells from ART naive patients. (**A**) ROS production by high-ROS lymphocytes (r = 0.355, p = 0.029, n = 38). (**B**) ΔΨm of high-ΔΨm lymphocytes (r = −0.417, p = 0.007, n = 40). (**C**) Percentage of low-ΔΨm lymphocytes (r = 0.457, p = 0.003, n = 40). (**D**) ROS production by high-ROS monocytes (r = 0.164, p = 0.288, n = 44) (**E**) ΔΨm of monocytes (r = −0.455, p = 0.003, n = 40).

ROS production by high-ROS lymphocytes positively correlated with the viral load (Figure 4A) while no correlation was found in monocytes (Figure 4D). The ΔΨm of high-ΔΨm lymphocytes decreased with viral load (Figure 4B), whereas the percentage of low-ΔΨm lymphocytes simultaneously increased (Figure 4C). A similar correlation was found between monocyte ΔΨm and viral load (Figure 4E).

### Discriminant Analysis Combining Three Morphological Parameters Revealed Differential Mitochondrial Network Responses to HIV-1 Infection and to ART in Lymphocytes and Monocytes

Morphological parameters were measured by stereological analysis of confocal photomicrographs after image analysis processing (Figure S3).

**Figure 5:**
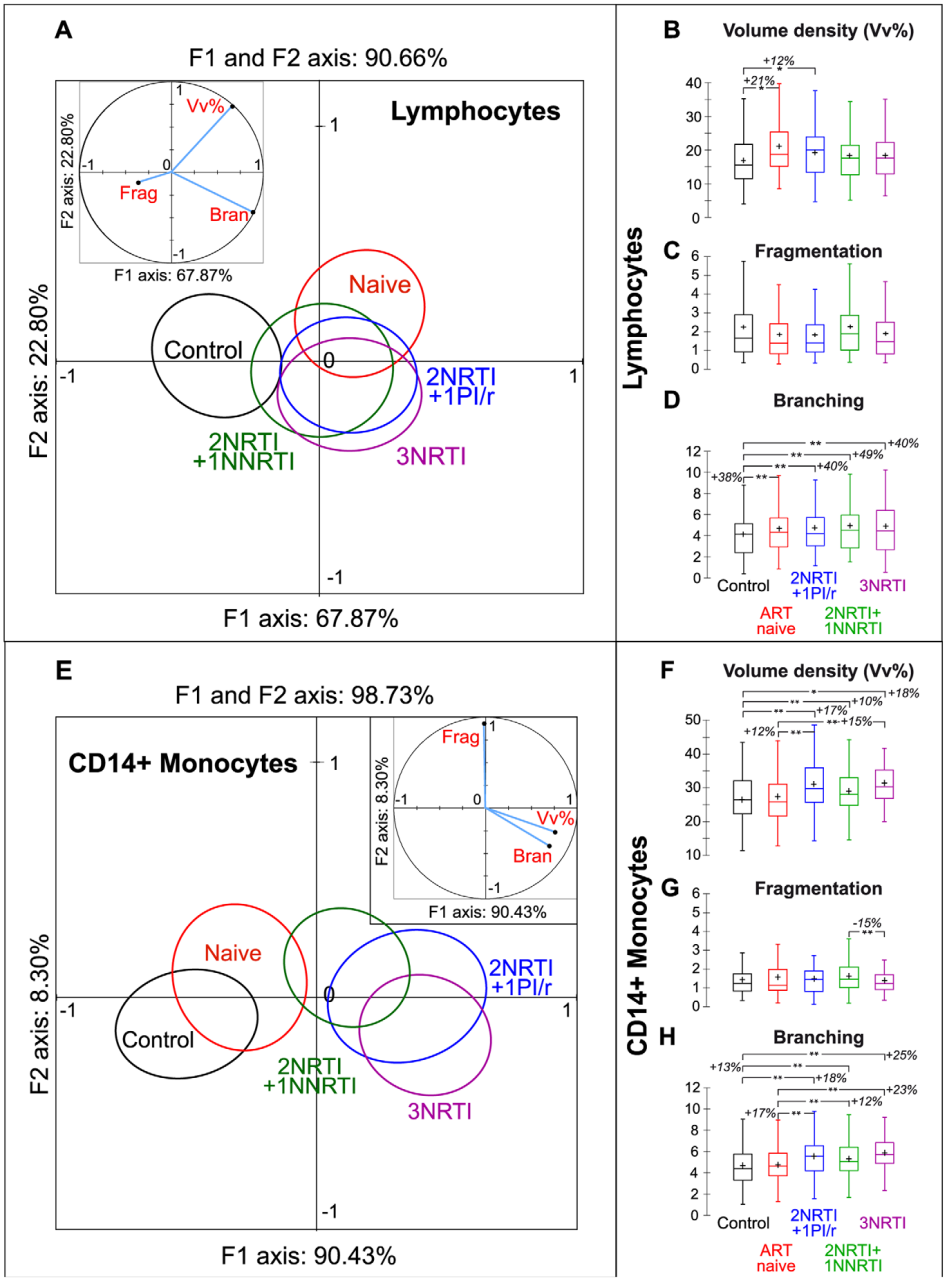
Mitochondrial morphological parameters: differential effects of HIV-1 infection and ART on lymphocytes and monocytes. (**A, E**) Discriminant analysis using three mitochondrial morphological parameters. The five cohort populations are delineated by their 95% confidence circles around the means. The two perpendicular axes describe the combined variance of the parameters analysed (lymphocytes: 90.66%, monocytes: 98.73%). The contributions of each parameter to the variances on the X and Y axes are shown in the insets. Inset: The 3 mitochondrial parameters used were: Vv%: Volume density (lymphocytes: see Figure 5B; monocytes: see Figure 5F). Frag: Fragmentation (lymphocytes: see Figure 5C; monocytes: see Figure 5G). Bran: Branching (lymphocytes: see Figure 5D; monocytes: see Figure 5H). (**A**) Lymphocytes ART naive patients and control subjects are statistically different. Mitochondrial changes induced by HIV infection are partially reduced by ART. (**E**) Monocytes ART naive patients exhibit no variations compared to control subjects, while significant differences are observed for the three ART groups. (**B to D; F to H**) Box plots: *p<0,05, **p<0,01.

Lymphocyte mitochondrial networks from control subjects were statistically different to those from ART naive patients (p = 0.002) and those from each ART group (2NRTI+1NNRTI: p = 0.027; 2NRTI+1PI/r: p = 0.011; 3NRTI: p = 0.009). However, no statistical difference was observed by comparison of ART naive patients with each ART group, or by comparison of the three ART groups, as shown by the close superposition of circles representing each patient group (Figure 5A).

In the ART naive patients, changes were mainly observed in volume density (Figure 5B) and branching (Figure 5D), but not fragmentation (Figure 5C). The ART combinations reversed the network volume density but failed to recover network branching.

In contrast, the same parameters either in isolation (Figures 5F to 5H), or in combination through discriminant analysis (Figure 5E), were similar in monocytes from ART naive patients and control subjects (p = 0.344). Interestingly, each ART population was statistically different from both control subjects (2NRTI+1NNRTI: p = 0.0002; 2NRTI+1PI/r and 3NRTI: p<0.0001) and ART naive patients (2NRTI+1NNRTI: p = 0.039; 2NRTI+1PI/r: p = 0.002; 3NRTI: p = 0.0001), as shown by the ART-induced increases in network volume density and branching. The three ART groups were not statistically different (p = 0.084).

### Demonstration of Qualitative, but not Quantitative, Changes in Three PBMC Mitochondrial Proteins by Western Blotting

The amounts of porin (a mitochondrial protein loading control), CoxIV-2 (encoded by the mitochondrial genome) and CoxIV-4 (encoded by the nuclear genome) purified from PBMCs were analysed relative to GAPDH in all cohort participants (Figure S5A). No statistically significant variation in the amounts of the three proteins was observed by either discriminant analysis combining the measurements of all three proteins (Figure S5B), or by comparing the amounts of the individual proteins (Figure S5C to E). Differences in the CoxIV-4 to CoxIV-2 ratio were also not observed (not shown).

A 29 kDa band corresponding to CoxIV-4 was detected in 21% of the ART naive patients, 30% of the 2NRTI+1NNRTI patients, and 36% of the 3NRTI patients. However, this band was detected in only 1 of 38 PBMC homogenates from control subjects, and in 1 of 28 2NRTI+1PI/r patients (Figure S5F). Co-labelling with two specific antibodies suggested that this band may correspond to monoubiquitinylated CoxIV-4 (not shown). Finally, bands corresponding to a defect in the cleavage of the CoxIV-4 mitochondrial targeting signal in PBMCs from patients treated with 2NRTI+1PI/r were not observed (Figure S5G).

## Discussion

The goal of the ANRS EP45 “Aging” study is to analyse, in a large cohort of HIV-1 infected and uninfected subjects, the impact of HIV-1 infection and of antiretroviral regimen on mitochondrial parameters.

### HIV-1 Infection Clearly Impacted Lymphocyte Mitochondria While Monocyte Mitochondria Appeared to be Less Sentitive

PBMC mitochondrial parameters from ART naive patients compared to control subjects were used to delineate the consequences of HIV-infection, including the effects of viral proteins [5], miRNAs [6], immunosenescence [30] and the innate immune response [3].

Western blot analysis showed a minor 29 kDa CoxIV-4 band in 21% of ART naive patients, which may have represented mono-ubiquitined CoxIV-4. Confirmation by co-immunoprecipitation experiments could not be performed because of the large amount of protein required. Mitochondrial protein mono- or poly-ubiquitinylation [31] may be the first step of mitophagy [32] or of protein proteasomal destruction [33]. Some E3 ubiquitin ligases have been localized in the OMM [34].

No variations in the amounts of the mitochondrial proteins, porin, CoxIV-2 and CoxIV-4, relative to GAPDH, were observed in PBMCs from ART naive patients compared to control subjects. Because protein assays were performed on total PBMC lysates, no distinction could be made between lymphocytes or monocytes, or between cell subgroups defined by their ROS production and ΔΨm. Thus, the variations between the different cell types may be masked. Nonetheless, proteome analysis in a cohort with similar clinical characteristics to ours showed that, compared to healthy donors, PBMCs from ART naive patients exhibited decreased levels of some Complex I (3), IV (5a) and ATP synthase (alpha) subunits but not of CoxIV-2 or CoxIV-4 [35]. Therefore, changes in subunits other than the two studied may result in abnormalities of ROS-producing complexes I and III [36], [37], and result in increased ROS production [35].

Mitochondrial functional investigations were performed on lymphocytes and moncytes separately. Lymphocyte FSC/SSC gate included various lymphocyte subpopulations such as T lymphocytes (≈ 80% of blood lymphocytes; CD3+ CD4+ and CD3+ CD8+ ≈ 40% each), B lymphocytes (CD3− CD19+ ≈ 4%) and NK cells (CD3− CD19−CD16+ CD56+ ≈ 3%) as shown by a ancillary study we performed. Our lymphocyte FSC/SSC gate may also contain dendritic cells present in blood (0.3 - 0.5% of PBMC) [38].

Flow cytometry identified two lymphocyte and CD14+ monocyte subgroups characterized by either high- or low-ROS production. While high- and low-ROS producing monocytes have been described previously [39], [40], the identification of lymphocytes that produce either high or low amounts of ROS is novel. The ancillary study, we performed, further characterised lymphocyte subtypes through their ROS production. T lymphocytes and B lymphocytes exhibited the same ROS pattern as the whole FSC/SSC-gated lymphocyte population. In contrast, NK cells were a pure population of low-ROS cells only. ROS production may therefore be another parameter that differentiates NK cells from T and B lymphocytes. Indeed, ROS are known to be physiological messengers in T and B cells [41].

IMM permeability, which is measured by ΔΨm, and ROS production are two related parameters [12]. ΔΨm measurements also indicated the presence of two statistically different groups of lymphocytes, as described for lymphocytes [24] and PBMCs [42]. As discussed previously, there were no relationships between ROS and the ΔΨm subgroups.

Discriminant analysis combining ROS and ΔΨm measurements better emphasized the mitochondrial differential effects of HIV-1 infection on the two peripheral blood cell types than comparison of single parameters. While lymphocyte mitochondria were statistically different in ART naive patients compared to control subjects, monocyte mitochondria showed no differences. HIV infection has previously been shown to induce general oxidant stress associated with a decrease in both lymphocyte and blood serum oxidant defence systems [43]. In addition, a decreased ΔΨm was previously reported in PBMCs from ART naive patients exhibiting a slightly lower CD4+ cell count than our patients [44], [45]. This decrease could be linked to HIV-1-encoded Vpr through its binding to mitochondrial permeability transition pore components [46].

Significant correlations between functional parameters and viral load further underlined the effect of HIV-1 infection on lymphocyte mitochondria. Numerous events may result in mitochondrial impairment in both infected cells and uninfected bystander cells through various signaling events. Indeed, ROS production by NADPH oxidase has been observed in uninfected bystander CD4+ lymphocytes after binding of the HIV-1 protein gp41 to a plasma membrane receptor [47]. Through the secretion of cytokines [48] or of viral proteins [49], infected macrophages or CD4+ T lymphocytes modified various functions of neighboring non-infected cells [50]. Additional interactions between infected and bystander T cells involved exocytosis and uptake of HIV-encoded Nef-bearing exosomes [51], [52]. Exosomes are also known to carry miRNAs, some of which are encoded by the HIV genome and are suspected to modifiy gene expression in recipient cells [53]. “Tunneling nanotubes” were also reported to carry viruses and viral proteins between infected macrophages and B lymphocytes [54].

Decreased ATP supply due to increased ROS production and lowered ΔΨm have been reported to result in mitochondrial network fragmentation, mitophagy, or cell apoptosis [13]. Morphological quantitative analysis of mitochondrial networks in lymphocytes from our ART naive patients argued against these events. These cells exhibited an increase in mitochondrial volume density and branching, whereas mitochondrial network fragmentation did not change. These observations exclude both mitophagy and subsequent lymphocyte apoptosis, and may rather reflect a primary cell response to oxidative stress [55] and/or to HIV-1 proteins [5], in addition to the triggering of innate immunity [56], [57].

In summary, HIV-1 infection in ART naive patients with a controlled CD4+ cell count and viral load resulted in significant alterations in four of the lymphocyte mitochondrial parameters (ROS production, ΔΨm, network volume density, branching), without irreversible damage that may lead to mitophagy and/or apoptosis.

In contrast to lymphocytes and previously published ROS data [58], mitochondria from monocytes did not exibit changes in functional or morphological parameters. Our results also contrasted with data from peripheral blood monocytes infected *in vitro* with HIV-1 and cultured for 7 days [59]. Nevertheless, monocyte sensitivity to HIV-1 infection was detected through correlation of one of the functional parameters (ΔΨm) and viral load in ART naive patients. Regarding the central role of ROS production via NADPH oxidase in phagocytic cells as a defence mechanism against infection [60], it is of interest to note that in resting monocytes, mitochondria use more oxygen (70%) than plasma membrane enzymes (30%) [61].

Our study of ART naive patients who were considered stable according to their clinical parameters, highlighted that HIV-1 infection did not affect monocyte mitochondria as much as it directly or indirectly impaired lymphocyte mitochondria, leading to minor oxidant stress known to favour viral replication, immune dysfunction and disease progression [62], [63].

### ART Partially Rescued HIV-1 Infection-induced Mitochondrial Abnormalities in Lymphocytes but Led to Mitochondrial Changes in Monocytes

Three different ART combinations did not modify the amounts of porin, CoxIV-2 and CoxIV-4, or the CoxIV-4/CoxIV-2 ratio, as previously reported [64], [65]. Nonetheless, conflicting data on the mtDNA content (and coxIV-2 mRNA or protein) in PBMCs have been reported in treated patients from cohorts exhibiting heterogeneous characteristics (e.g. CD4+ cell count, viral load.) [66], [67], [68]. In skeletal muscle, NRTIs have been shown to increase the frequency of the mtDNA Δ4977 “common deletion”, leading to a CoxIV complex biosynthesis defect and enzymatic deficiency [27]. The lack of CoxIV protein abnormalities in our study could be explained by the large difference in lifespan between PBMCs and skeletal muscle, and/or by the fact that PBMC mitochondria may be less sensitive to ART side-effects than other uninfected tissues (e.g. skeletal muscle, adipose tissue), as shown for mtDNA [69], [70]. In addition, a PI-containing regimen did not inhibit mitochondrial protease(s) involved in the cleavage of the mitochondrial targeting signal in nuclear-encoded CoxIV-4 isoforms 1 or 2 [71], because we did not observe a 20 kDa band that corresponded to uncleaved proteins. Thus, the biosynthesis and maturation of PBMC proteins encoded either by nuclear or mitochondrial genomes from our cohort patients were not impaired. The CoxIV-4 29 kDa band observed in PBMCs from some ART naive patients was also detected in PBMCs from some patients under 2NRTI+1NNRTI and 3NRTI. If this band represented mono-ubiquitined CoxIV-4, the lack of its detection in PBMCs from patients treated with a PI/r-containing ART combination could argue against the fact that PI inhibits proteasomal activity, as postulated from *in vitro* studies [72], [73].

Discriminant analyses integrating functional and morphological mitochondrial parameters revealed another discrepancy between lymphocytes and monocytes in response to ART. While ART regimens partially improved lymphocyte mitochondrial parameters, none of the ART combinations fully restored mitochondrial changes induced by HIV-1 infection. In monocytes, the mitochondrial parameters remained unchanged in ART naive patients whereas the three ART regimens resulted in slight (functional) or significant (morphological) modifications. Previous studies have shown either ART-induced ΔΨm recovery [24], [44] or impairment [74] in lymphocytes or PBMCs. Heterogeneous data regarding mitochondrial networks were also reported. Under the NRTI regimen, mitochondrial mass/volume has been shown to decrease in PBMCs [75], to be unchanged in PBMCs [76] or CD4+ T cells [77], and to be increased in CD8+ T cells [78]. However, NRTI induced a mitochondrial mass/volume increase in cultured human adipocytes [79], in a cultured hepatocyte cell line [80], and in subcutaneous adipocytes from patient biopsies [81].

None of the three first line ART regimens appeared to be more toxic towards PBMC mitochondria than the others. Moreover, the majority of 2NRTI+1PI/r and 2NRTI+1NNRTI patients shared the same NRTI backbone. Because no statistical difference was found between these two populations using discriminant analysis, our data suggested that boosted Lopinavir (68% of PI) and Efavirenz (72% of NNRTI) did not elicit major mitochondrial effects. The ART regimens in our patient cohort mainly included drugs of the second generation, whose mitochondrial toxicity is reduced in comparison with first generation drugs [82]. As for HIV-1 infection, the increases in volume density and branching parameters induced by ART combinations could be interpreted as responses to mitochondrial stress [55], the result of immunosenescence [30], and/or the result of an innate immune response [83], [84].

### Several Factors could Explain the Mitochondrial Differences between Lymphocytes and Monocytes

In contrast to lymphocytes, HIV-1 infects very few blood monocytes (less than 1%) as shown by the lower proviral DNA copy number in monocytes than in CD4+ T cells. Moreover, blood monocytes supported only a very low viral replication rate, as shown by the detection of 2 LTR circles, HIV mRNAs, or changes with time in viral nucleotide sequences. Monocyte migration out of the blood into tissues, followed by differentiation into macrophages or dendritic cells, increased the inhibition of viral replication (reviewed in [85], [86], [87]). HIV-1 Vpx protein has been demonstrated to relieve replication restriction in monocytes [88] by inducing the proteasomal degradation of SAM domain and HD domain-containing protein 1 (SAMHD1), the HIV-1 restriction factor that is specific for myeloid lineage cells and is not expressed by lymphocytes [89]. Despite their short (≈ 72 h) blood circulation time [90], monocytes may become infected as bone marrow precursors [91] or later in the blood, and may contribute to the viral reservoir [92] together with bone marrow stem cells [93]. Correlation between monocyte ΔΨm and viral load could argue in favour of monocyte sensitivity to viral replication, thus demonstrating the limit of restriction defence systems.

In lymphocytes but not monocytes, the negative side-effects of ART (mitotoxicity) on mitochondria could be balanced by the positive effects of ART, i.e. decreased viral load and replication [94]. ART antiviral activity is less effective towards chronically infected monocytes than lymphocytes for several reasons, one of them being that HIV-related ART targets are present in lower amounts in refractory monocytes [95]. Moreover, the different mitochondrial sensitivities to ART could also be the result of distinct pathways involved in ART intracellular metabolism (e.g. influx, efflux, degradation) within the two cell types [96].

Taken together, these data suggest that mitochondria from blood lymphocytes and monocytes exhibit differential responses to either HIV-1 infection or ART. Whereas mitochondrial ROS and ΔΨm functional parameters exhibited changes, mitochondrial morphological modifications argued against apoptosis [9] or mitophagy [32] induced by either HIV-1 infection or ART regimens. These mitochondrial changes could be related to innate immunity signalling pathways through ROS- and ΔΨm-regulated MAVS [3], [19]. Both direct and bystander effects are associated with HIV-1 infection [51] clearly impacted lymphocyte mitochondria while monocyte mitochondria appeared to be less sentitive. First line ART tended to rescue lymphocyte mitochondrial parameters altered by viral infection but induced only slight changes in monocytes. Besides the slight alterations reported in our patients, who were considered to be clinically stable, evolution and/or persistent changes overtime in PBMCs and other tissues may contribute to body aging. Indeed, physiological aging is known to correlate with the decline in mitochondrial respiratory function and with ROS overproduction [97].

## Supporting Information

Figure S1 **Cell viability analysis.** Representative density plots of peripheral blood lymphocytes (**A**) and CD14+ monocytes (**B**) from an HIV-1 infected patient. Blood collected in EDTA Vacutainer tubes was rotated overnight prior to PBMC isolation. Cell viability was determined after PBMC incubation for 3 h at 37°C, 5% CO_2_ in RPMI medium supplemented with 10% fœtal bovine serum and 2 mM L-glutamine. Dead cells were double stained with 7AAD and annexin V, and early apoptotic cells were identified by annexin V staining only. Viability remained over 97%. Apoptotic cell numbers were negligible. Control cells incubated overnight with 1 µM staurosporin were used as a positive control for apoptosis (lymphocytes, **C**; monocytes, **D**).
(TIF)

Figure S2 **Correlation between ROS production and ΔΨm in lymphocyte mitochondria.** Negative correlation between basal ROS production and ΔΨm (r = −0.318, p<0.001). Total lymphocyte population from all cohort participants (n = 157).
(TIF)

Figure S3 **Morphological analysis of the monocyte mitochondrial network.** The mitochondrial network was imaged using confocal microscopy (30% argon laser stimulation; PMT set on standard preregistered Mitotracker fluorescence emission spectrum; 100×magnification; 512×512 pixels; speed 400 Hz; zoom factor 9; line average: 3; slice thickness: 0.05 µm). One single slice was recorded for each monocyte identified using DAPI nuclear stain. 30 pictures were concatenated and analysed using a specifically designed Image J macro (available on request). The cell surface area and the mitochondrial network area were measured after automatic thresholding and binarization. Mitochondrial network images were then skeletonised, and skeleton connectivity was determined as follows: isolated pixels and pixels with only one neighbour were numbered (network ends); pixels with two neighbours were used for network length and fragmentation measurement; and pixels with three or more neighbours were used for the estimation of network branching. Data were stored in Excel files. The volume density of the mitochondrial network was calculated as the ratio of mitochondrial network surface area to monocyte surface area and expressed as a percentage. Fragmentation was estimated as the ratio of the number of skeleton fragments of the mitochondrial network to the total length of the skeleton. Branching was determined as the ratio of the number of pixels having three or more neighbours to the total skeleton length. The flow cytometric FSC parameter showed that the mean volume of both lymphocytes and monocytes was not statistically different among the 5 patient groups. This allowed for comparison between the stereological parameters related to lymphocyte or monocyte mean volume. Image processing of 4 monocytes exhibiting different representative morphological characteristics. From top to bottom: (**A**)**,** (**D**)**,** (**G**)**,** (**J**) Original Mitotracker confocal slide pictures (**B**)**,** (**E**)**,** (**H**)**,** (**K**) Binary pictures of the mitochondrial network after thresholding (**C**)**,** (**F**)**,** (**I**)**,** (**L**) Mitochondrial network skeleton (white line) Isolated pixels are highlighted in blue, skeleton end pixels are highlighted in red, yellow points indicate pixels with three or more neighbours and correspond to branching points. (Bar = 2 µm)
(TIF)

Figure S4 **ROS production by lymphocyte subtypes.** Representative graphs for HIV-1 infected patients. Lymphocyte subtypes were stained using specific CD antibodies (Beckman Coulter Inc) after treatment with ROS according to a standard procedure. 20,000 FSC/SSC-gated lymphocytes were analysed using a Navios flow cytometer (Beckman Coulter Inc). Two panels were used to identify lymphocyte subpopulations, one for T subtypes and a second for B and NK lymphocytes. Overlays of dot plots (left panels; **A, C, E, G, I**) and histograms (right panels; **B, D, F, H, J**) of ROS production by the total lymphocyte population (gray) and lymphocyte subtypes (red) were reported. ROS MFI ± SD are shown for each population. The percentages were related to the total lymphocyte population (FSC/SSC). (**A, B**) CD3+ T lymphocytes (**C, D**) CD3+ CD4+ T lymphocytes (**E, F**) CD3+ CD8+ T lymphocytes (**G, H**) CD3− CD19+ B lymphocytes (**I, J**) CD3− CD19− CD16+ CD56+ NK lymphocytes CD3− CD19+ B, CD3+ CD4+ and CD3+ CD8+ T lymphocytes, representing the large majority of lymphocytes, exhibited the same pattern as the total lymphocyte population with both low- and high-ROS producing cells, whereas CD3− CD19− CD16+ CD56+ NK lymphocytes were defined as low-ROS producing cells only.
(TIF)

Figure S5 **Western blot analysis of PBMC mitochondrial proteins.** (**A**) Representative western blot: porin (mitochondrial protein loading control, 31 kDa), CoxIV-2 (encoded by the mitochondrial genome, 20 kDa) and CoxIV-4 (encoded by the nuclear genome, 15 kDa). GAPDH was used as a total protein loading control (37 kDa). Participants were age- and sex-matched. No variation in protein amounts was observed between the participant groups. Human alveolar macrophages were used as a control for antibodies. (**B**) Discriminant analysis using three PBMC mitochondrial proteins. The five cohort populations are delineated by their 95% confidence circles around the means. The two perpendicular axes describe the combined variance (97.45%) of the parameters analysed. The inset shows the contribution of the amounts of each protein to the variance associated with X and Y axes. No statistical differences are observed between the groups. (**C to E**) Box plot of PBMC mitochondrial protein comparison from the five cohort groups: porin (C), CoxIV-2 (D) and CoxIV-4 (E). No statistical differences are observed between the groups (p = 0,93). (**F**) Western blot showing a faint 29 kDa CoxIV-4 positive band in some patients. (**G**) Western blot showing an absence of bands corresponding to a defect in the cleavage of CoxIV-4 mitochondrial targeting signal in patients treated with 2NRTI+1PI/r.
(TIF)

Table S1 **Demographic, clinical and biological parameters of the subjects/patients in the ANRS EP45 “Aging” cohort.** No differences with respect to age and sex were present in the five groups. CD4+ cell count, CD4+/CD8+ and viral load indicated that the HIV-1 infected patients could be considered clinically stable.
(DOC)

Table S2 **Details of the ART treatments.** Three ART combinations (2NRTI+1PI/r; 2NRTI+1NNRTI; 3NRTI) were used in accordance with World Health Organisation and French Health Ministry current recommendations. 70% of the 2NRTI+1PI/r and 2NRTI+1NNRTI patients shared the same Tenofovir/Emtricitabine NRTI backbone, and 93% of the 3NRTI patients shared a Lamivudine/Zidovudine NRTI backbone. Lopinavir/r and Efavirenz were the main PI/r (68%) and NNRTI (72%) used, respectively.
(DOC)

Protocol S1 **The ANRS EP45 “Aging” Study.** http://clinicaltrials.gov/, NCT01038999.
(PDF)

Methods S1 **Cell viability and western blot procedures.**
(DOC)
