# Supplementary figures and images for: HIV-1 Infection and First Line ART Induced Differential Responses in Mitochondria from Blood Lymphocytes and Monocytes: The ANRS EP45 “Aging” Study
Source: PLoS One. 2012 Jul 19;7(7):e41129. doi: 10.1371/journal.pone.0041129 (PMC3400613; doi:10.1371/journal.pone.0041129)

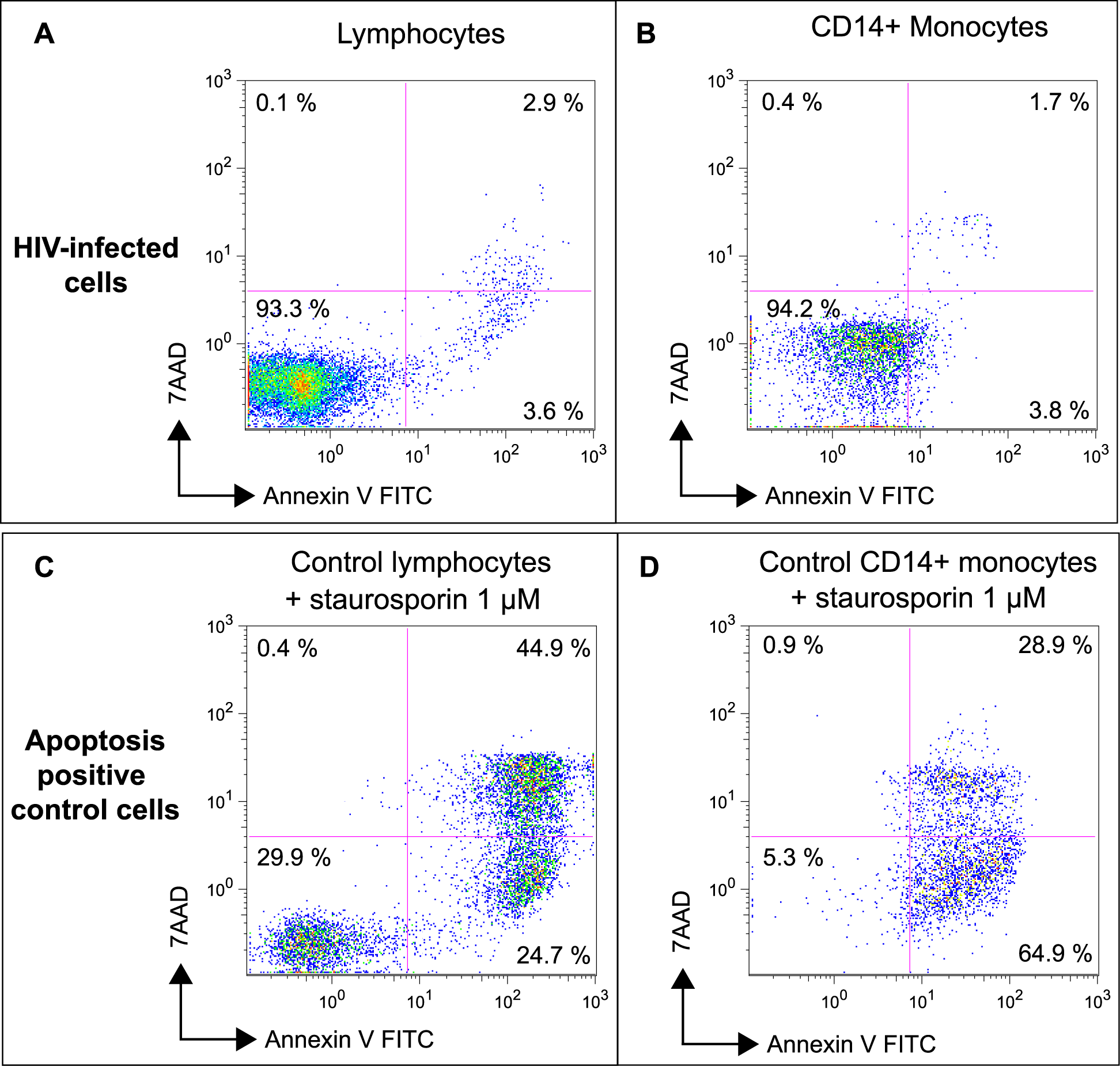

Supplement: Figure S1 — Cell viability analysis. Representative density plots of peripheral blood lymphocytes (A) and CD14+ monocytes (B) from an HIV-1 infected patient. Blood collected in EDTA Vacutainer tubes was rotated overnight prior to PBMC isolation. Cell viability was determined after PBMC incubation for 3 h at 37°C, 5% CO2 in RPMI medium supplemented with 10% fœtal bovine serum and 2 mM L-glutamine. Dead cells were double stained with 7AAD and annexin V, and early apoptotic cells were identified by annexin V staining only. Viability remained over 97%. Apoptotic cell numbers were negligible. Control cells incubated overnight with 1 µM staurosporin were used as a positive control for apoptosis (lymphocytes, C; monocytes, D). (TIF) [file pone.0041129.s001.tif]

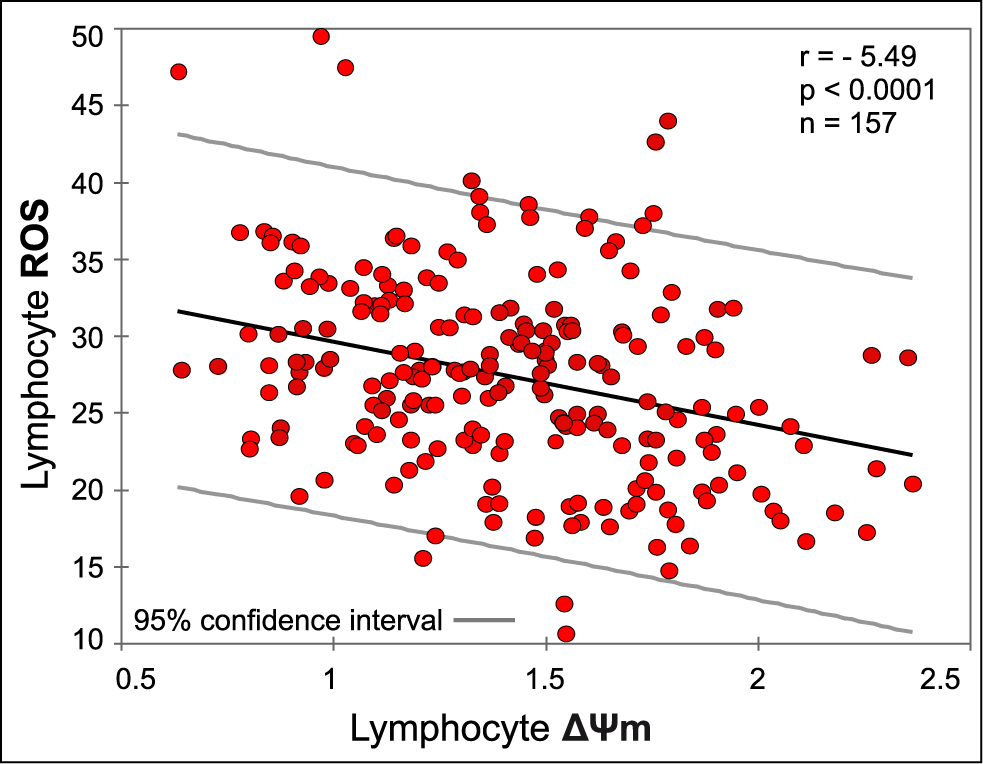

Supplement: Figure S2 — Correlation between ROS production and ΔΨm in lymphocyte mitochondria. Negative correlation between basal ROS production and ΔΨm (r = −0.318, p<0.001). Total lymphocyte population from all cohort participants (n = 157). (TIF) [file pone.0041129.s002.tif]

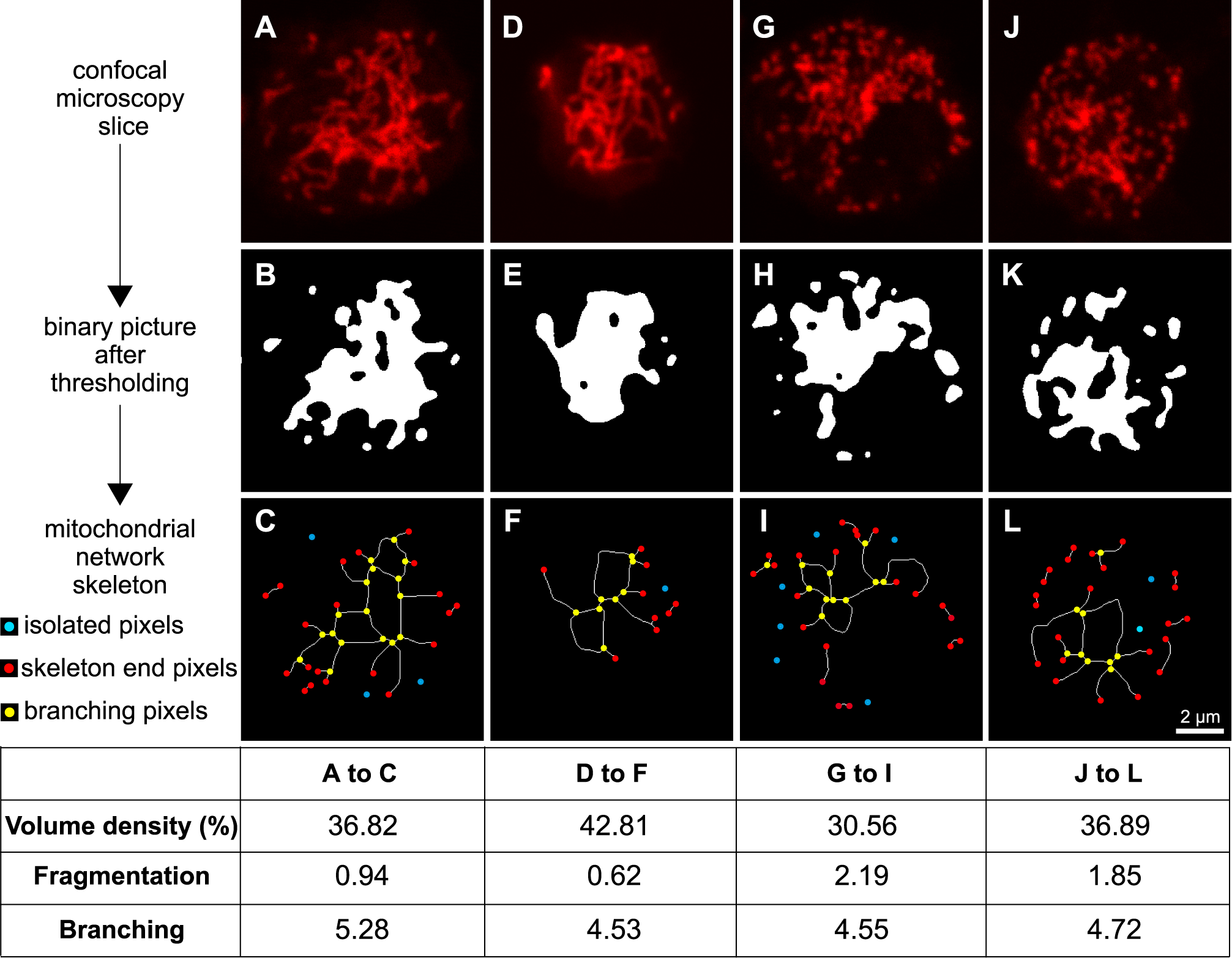

Supplement: Figure S3 — Morphological analysis of the monocyte mitochondrial network. The mitochondrial network was imaged using confocal microscopy (30% argon laser stimulation; PMT set on standard preregistered Mitotracker fluorescence emission spectrum; 100×magnification; 512×512 pixels; speed 400 Hz; zoom factor 9; line average: 3; slice thickness: 0.05 µm). One single slice was recorded for each monocyte identified using DAPI nuclear stain. 30 pictures were concatenated and analysed using a specifically designed Image J macro (available on request). The cell surface area and the mitochondrial network area were measured after automatic thresholding and binarization. Mitochondrial network images were then skeletonised, and skeleton connectivity was determined as follows: isolated pixels and pixels with only one neighbour were numbered (network ends); pixels with two neighbours were used for network length and fragmentation measurement; and pixels with three or more neighbours were used for the estimation of network branching. Data were stored in Excel files. The volume density of the mitochondrial network was calculated as the ratio of mitochondrial network surface area to monocyte surface area and expressed as a percentage. Fragmentation was estimated as the ratio of the number of skeleton fragments of the mitochondrial network to the total length of the skeleton. Branching was determined as the ratio of the number of pixels having three or more neighbours to the total skeleton length. The flow cytometric FSC parameter showed that the mean volume of both lymphocytes and monocytes was not statistically different among the 5 patient groups. This allowed for comparison between the stereological parameters related to lymphocyte or monocyte mean volume. Image processing of 4 monocytes exhibiting different representative morphological characteristics. From top to bottom: (A), (D), (G), (J) Original Mitotracker confocal slide pictures (B), (E), (H), (K) Binary pictures of the mitochondrial ne [file pone.0041129.s003.tif]

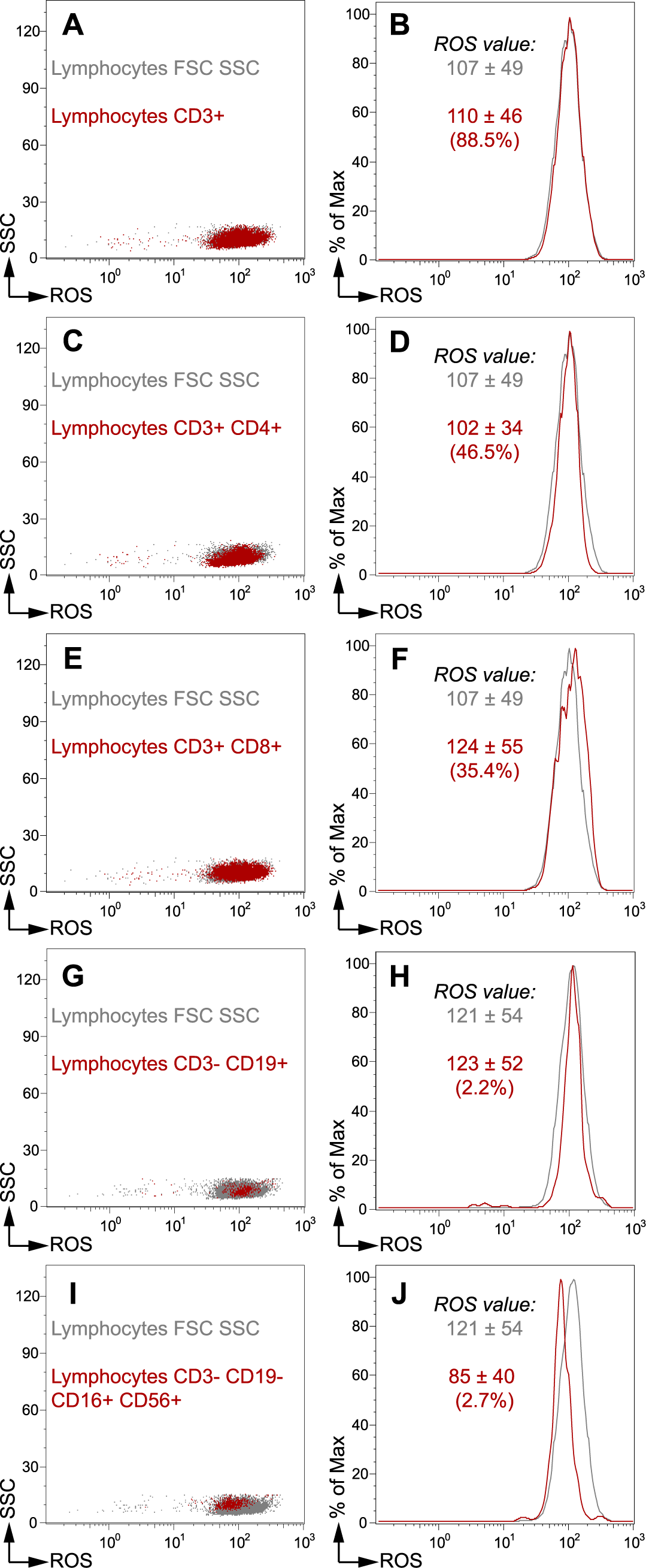

Supplement: Figure S4 — ROS production by lymphocyte subtypes. Representative graphs for HIV-1 infected patients. Lymphocyte subtypes were stained using specific CD antibodies (Beckman Coulter Inc) after treatment with ROS according to a standard procedure. 20,000 FSC/SSC-gated lymphocytes were analysed using a Navios flow cytometer (Beckman Coulter Inc). Two panels were used to identify lymphocyte subpopulations, one for T subtypes and a second for B and NK lymphocytes. Overlays of dot plots (left panels; A, C, E, G, I) and histograms (right panels; B, D, F, H, J) of ROS production by the total lymphocyte population (gray) and lymphocyte subtypes (red) were reported. ROS MFI ± SD are shown for each population. The percentages were related to the total lymphocyte population (FSC/SSC). (A, B) CD3+ T lymphocytes (C, D) CD3+ CD4+ T lymphocytes (E, F) CD3+ CD8+ T lymphocytes (G, H) CD3− CD19+ B lymphocytes (I, J) CD3− CD19− CD16+ CD56+ NK lymphocytes CD3− CD19+ B, CD3+ CD4+ and CD3+ CD8+ T lymphocytes, representing the large majority of lymphocytes, exhibited the same pattern as the total lymphocyte population with both low- and high-ROS producing cells, whereas CD3− CD19− CD16+ CD56+ NK lymphocytes were defined as low-ROS producing cells only. (TIF) [file pone.0041129.s004.tif]

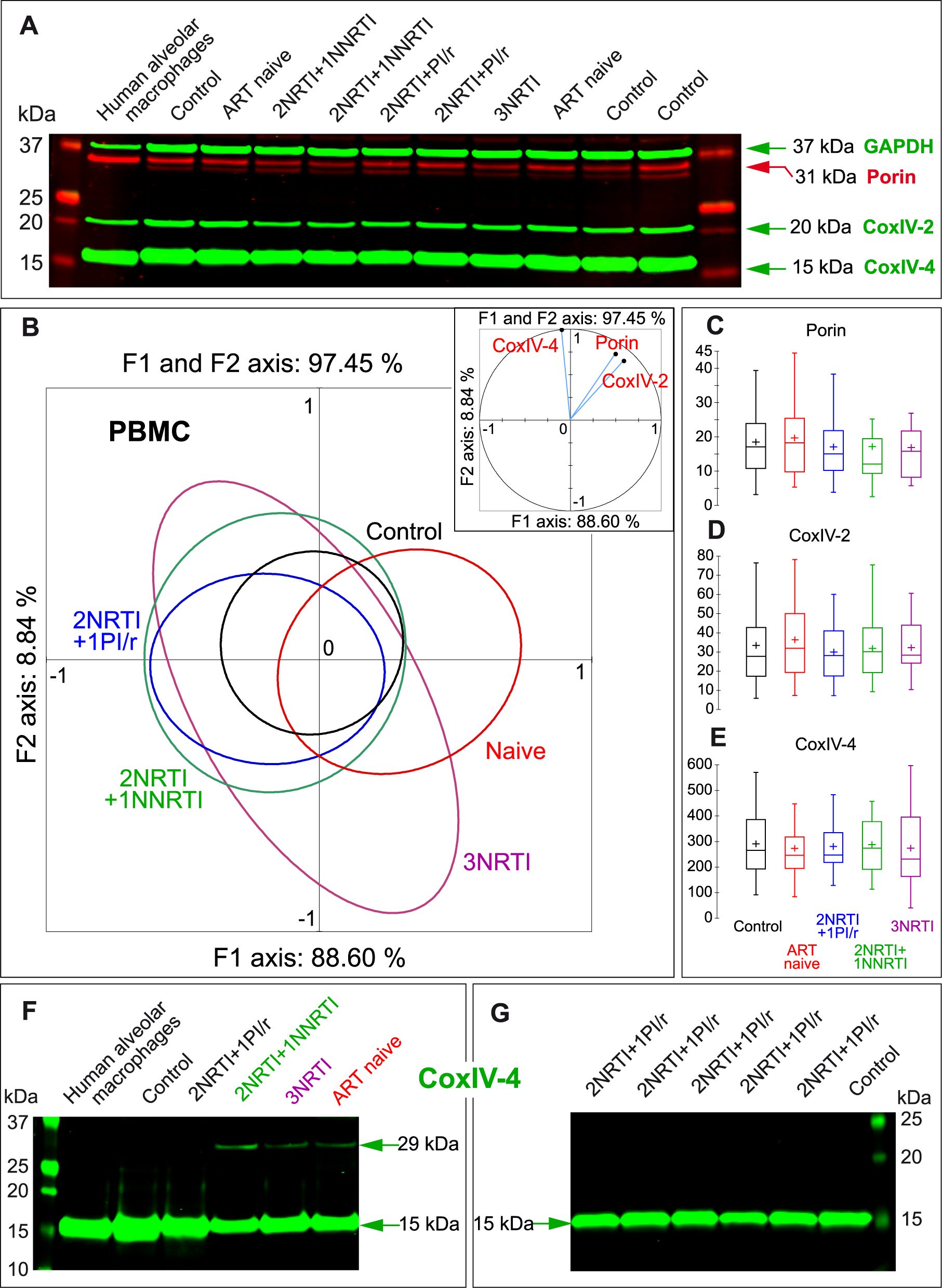

Supplement: Figure S5 — Western blot analysis of PBMC mitochondrial proteins. (A) Representative western blot: porin (mitochondrial protein loading control, 31 kDa), CoxIV-2 (encoded by the mitochondrial genome, 20 kDa) and CoxIV-4 (encoded by the nuclear genome, 15 kDa). GAPDH was used as a total protein loading control (37 kDa). Participants were age- and sex-matched. No variation in protein amounts was observed between the participant groups. Human alveolar macrophages were used as a control for antibodies. (B) Discriminant analysis using three PBMC mitochondrial proteins. The five cohort populations are delineated by their 95% confidence circles around the means. The two perpendicular axes describe the combined variance (97.45%) of the parameters analysed. The inset shows the contribution of the amounts of each protein to the variance associated with X and Y axes. No statistical differences are observed between the groups. (C to E) Box plot of PBMC mitochondrial protein comparison from the five cohort groups: porin (C), CoxIV-2 (D) and CoxIV-4 (E). No statistical differences are observed between the groups (p = 0,93). (F) Western blot showing a faint 29 kDa CoxIV-4 positive band in some patients. (G) Western blot showing an absence of bands corresponding to a defect in the cleavage of CoxIV-4 mitochondrial targeting signal in patients treated with 2NRTI+1PI/r. (TIF) [file pone.0041129.s005.tif]
