# Supplementary material for: HIV-1 Infection and First Line ART Induced Differential Responses in Mitochondria from Blood Lymphocytes and Monocytes: The ANRS EP45 “Aging” Study
Source: PLoS One. 2012 Jul 19;7(7):e41129. doi: 10.1371/journal.pone.0041129 (PMC3400613; doi:10.1371/journal.pone.0041129)
Supplement: Table S1 — Demographic, clinical and biological parameters of the subjects/patients in the ANRS EP45 “Aging” cohort. No differences with respect to age and sex were present in the five groups. CD4+ cell count, CD4+/CD8+ and viral load indicated that the HIV-1 infected patients could be considered clinically stable. (DOC) [file pone.0041129.s006.doc]

**Supporting information**

Perrin et al.: HIV-1 Infection and First Line ART Induced Differential Responses in Mitochondria from Blood Lymphocytes and Monocytes: the ANRS EP45 “Aging” Study.

**Supporting Tables**

**Supporting Table S1.** Demographic, clinical and biological parameters of the subjects/patients in the ANRS EP45 “Aging” cohort

| **Demographic information** | **Uninfected control subjects*** | **Group 1 (ART naive)** | **Group 2a (2NRTI**  **+1PI/r regimen)** | **Group 2b (2NRTI**  **+1NNRTI regimen)** | **Group 2c (3NRTI regimen)** | **p**** |
| --- | --- | --- | --- | --- | --- | --- |
| **n** | 49 | 49 | 35 | 32 | 14 | - |
| **Age (years)** | 39.9 ± 8.5 | 39.4 ± 8.0 | 40.7 ± 9.3 | 41.7 ± 9.3 | 44.4 ± 7.8 | 0.344 (AN) |
| **Men, n (%)** | 38 (77.6) | 38 (77.6) | 27 (77.1) | 27 (84.4) | 11 (78.6) | 0.944 (Fs) |
| **Years since HIV diagnosis** | - | 6.8 ± 6.1 | 5.4 ± 5.4 | 8.9 ± 7.7 | 9.9 ± 5.5 | 0.003 (KW)  *0.002 (KW)* |
| **CDC clinical stage** |  |  |  |  |  | 0.085 (Fs)  *0.695 (Fs)* |
| A, n (%) | - | 43 (87.8) | 23 (65.7) | 26 (81.3) | 11 (78.6) |  |
| B, n (%) | - | 6 (12.2) | 7 (20.0) | 3 (9.4) | 2 (14.3) |  |
| C, n (%) | - | 0 | 5 (14.3) | 3 (9.4) | 1 (7.1) |  |
| **CD4+ cell count (cells/mm^3^)** | - | 548.4  ± 220.0 | 561.1  ± 181.7 | 592.2  ± 232.7 | 741.9  ± 246.5 | 0.052 (KW)  *0.059 (KW)* |
| **CD4+/CD8+ ratio** | - | 0.63 ± 0.32 | 0.87 ± 0.45 | 0.92 ± 0.45 | 1.14 ± 0.52 | <.0001 (AN)  *0.178 (AN)* |
| **Viral load ≤ 40 copies/ml, n (%)** | - | 1 (2.0) | 31 (88.6) | 29 (90.6) | 13 (92.9) | <.0001 (K2)  *1.000 (Fs)* |
| **Log_10_ Viral load** | - | 3.8 ± 0.8 | 1.54 ± 0.29 | 1.64 ± 0.61 | 1.60 ± 0.18 | <.0001 (KW)  *0.397 (KW)* |
| **First line ART length (years)** | - | - | 2.7 ± 1.3 | 3.5 ± 2.5 | 7.3 ± 2.4 | <.0001 (KW) |

Mean ± SD. n: number of patients or of control subjects (%).

* Uninfected controls were age- and gender-matched with ART naive patients.

** p value for comparison of the five subjects/patients groups (or 4 patients groups) and p value for comparison of the 3 ART combinations (p value and statistical test italicized). (AN): Analysis of variance, (KW): Kruskal-Wallis test, (K2): Chi-square test, (Fs): Fisher exact test.
