# Supplementary material for: HIV-1 Infection and First Line ART Induced Differential Responses in Mitochondria from Blood Lymphocytes and Monocytes: The ANRS EP45 “Aging” Study
Source: PLoS One. 2012 Jul 19;7(7):e41129. doi: 10.1371/journal.pone.0041129 (PMC3400613; doi:10.1371/journal.pone.0041129)
Supplement: Table S2 — Details of the ART treatments. Three ART combinations (2NRTI+1PI/r; 2NRTI+1NNRTI; 3NRTI) were used in accordance with World Health Organisation and French Health Ministry current recommendations. 70% of the 2NRTI+1PI/r and 2NRTI+1NNRTI patients shared the same Tenofovir/Emtricitabine NRTI backbone, and 93% of the 3NRTI patients shared a Lamivudine/Zidovudine NRTI backbone. Lopinavir/r and Efavirenz were the main PI/r (68%) and NNRTI (72%) used, respectively. (DOC) [file pone.0041129.s007.doc]

**Supporting information**

Perrin et al.: HIV-1 Infection and First Line ART Induced Differential Responses in Mitochondria from Blood Lymphocytes and Monocytes: the ANRS EP45 “Aging” Study.

**Supporting Tables**

**Supporting Table S2.** Details of ART treatments

| **2NRTI Backbone (81)** |  | **Tenofovir + Emtricitabine (47)** | **Lamivudine + Zidovudine (27)** | **Lamivudine + Abacavir (6)** | **Lamivudine + Tenofovir (1)** |
| --- | --- | --- | --- | --- | --- |
| **NNRTI (32)** | Efavirenz | 20 | 1 | 1 | 1 |
|  | Nevirapine | 2 | 7 | - | - |
| **PI (35)** | Lopinavir | 16 | 5 | 3 | - |
|  | Fosamprenavir | 4 | - | 1 | - |
|  | Atazanavir | 4 | - | 1 | - |
|  | Saquinavir | 1 | - | - | - |
| **NRTI (14)** | Abacavir | - | 13 | - | - |
|  | Didanosine | - | 1 | - | - |
