# Supplementary material for: HIV-1 Infection and First Line ART Induced Differential Responses in Mitochondria from Blood Lymphocytes and Monocytes: The ANRS EP45 “Aging” Study
Source: PLoS One. 2012 Jul 19;7(7):e41129. doi: 10.1371/journal.pone.0041129 (PMC3400613; doi:10.1371/journal.pone.0041129)
Supplement: Protocol S1 — The ANRS EP45 “Aging” Study. http://clinicaltrials.gov/, NCT01038999. (PDF) [file pone.0041129.s008.pdf]

ClinicalTrials.gov
A service of the U.S. National Institutes of Health

Home Search Study Topics Glossary
Search

Full Text View | Tabular View | No Study Results Posted | Related Studies

Accelerated Aging, HIV Infection, Antiretroviral Therapies (EP 45)

This study is ongoing, but not recruiting participants.

First Received on December 23, 2009. Last Updated on September 12, 2011 History of Changes

**Sponsor:**	French National Agency for Research on AIDS and Viral Hepatitis
**Information provided by:**	French National Agency for Research on AIDS and Viral Hepatitis
**ClinicalTrials.gov Identifier:**	NCT01038999

Purpose

The main goal is to confirm, among HIV1-infected patients, data from in vitro studies showing that antiretroviral therapies induce an accelerated aging through the same mechanisms than genetic laminopathies or than "physiological " aging, that is through the synthesis and persistence of farnesylated prelamin A. The secondary goal is to measure the impact of HIV infection and of antiretroviral therapies on markers of cell ageing (proteasome, mitochondria, telomere). The perspective is to fix antiretroviral therapy side effects using the same drug combination that will be used in few weeks in Marseille to treat children suffering from progeria

Condition	Intervention
HIV Infection<br>Aging Accelerated<br>Antiretroviral Therapies	Biological: Peripheral blood biological tests

Study Type: Observational
Study Design: Observational Model: Case Control
Time Perspective: Prospective

Official Title: Accelerated Aging, HIV Infection, Antiretroviral Therapies

Resource links provided by NLM:

Genetics Home Reference related topics: complement factor I deficiency

MedlinePlus related topics: HIV/AIDS

U.S. FDA Resources

Further study details as provided by French National Agency for Research on AIDS and Viral Hepatitis:

Primary Outcome Measures:

- lamin A measurement by western blotting [Designated as safety issue: No]

Secondary Outcome Measures:

- Peripheral blood biological tests (cellular, molecular genetic) [Designated as safety issue: No]

Estimated Enrollment: 200
Study Start Date: April 2009
Estimated Study Completion Date: April 2013
Primary Completion Date: April 2010 (Final data collection date for primary outcome measure)

Groups/Cohorts	Assigned Interventions
A HIV1-infected naive patients	Biological: Peripheral blood biological tests<br>A group and B group will be evaluated three times, at baseline, then every 12 months during 3 years. In case of initiation or changing of antiretroviral therapy, patients will be evaluated once more. Control subjects will be only evaluated at baseline.
B HIV1-infected patients<br>in 1st line of ARV therapy for at least 12 months	Biological: Peripheral blood biological tests<br>A group and B group will be evaluated three times, at baseline, then every 12 months during 3 years. In case of initiation or changing of antiretroviral therapy, patients will be evaluated once more. Control subjects will be only evaluated at baseline.
C= control Non infected HIV volunters	Biological: Peripheral blood biological tests<br>A group and B group will be evaluated three times, at baseline, then every 12 months during 3 years. In case of initiation or changing of antiretroviral therapy, patients will be evaluated once more. Control subjects will be only evaluated at baseline.

Detailed Description:
Protease inhibitors block viral protease, as well as various other cell enzymes : ZMPSTE24 cliping off prelamin A into mature lamin A ; at least one of the Golgi proteases involved in the release of SREBP, controlling the transcription of lipid metabolism regulating genes ; mitochondrial proteases involved in the importation and further maturation of nuclear genome encoded proteins ; proteasome regulating the transcription of several genes through NF-B ; P450 cytochromes. Nucleosides inhibitors of the viral reverse transcriptase exhibit nuclear and mitochondrial DNA toxicity, disrupt lipid and protein glycosylation and inhibit telomerase. Therefore antiretroviral therapies target several pathways involved in accelerated or normal aging. Their combined effects are added to viral infection direct symptoms or to cell abnormalities induced by viral proteins.

Our multicentric (the 3 CISIH from Marseille, Nice and Montpellier) 3 year- long study will analyse 50 HIV1-infected naive patients (A group), appararied to 50 age- and sex-matched seronegative control subjects (recruited by CIC-UPCET of Marseille) and 100 HIV1-infected patients in first line of antiretroviral therapy for at least 12 months (B group). Patients of group A and B will be recruited in the 3 clinical unit. The HIV1- infected patients will be evaluated four times, at baseline, then every 12 months during 3 years. In case of initiation or changing of antiretroviral therapy, patients will be evaluated once more. Control subjects will be only evaluated at baseline.

Peripheral blood biological tests will be the following [Laboratory designation] : i/ viral load measurement, PBMC isolation, DNA extraction, proviral DNA measurement, cell and DNA storage [Virology, Timone CHU, Marseille]; ii/ assays of CD4, CD8, glycemia, insulinemia, HOMA, total-, LDL- and HDL-cholesterol, triglycerides [Biochemistry labs from the 3 CHU] ; iii/ antiretroviral drug assay (mass spectrometry) [Pharmacokinetics, Timone CHU, Marseille]; iv/ detection (western blotting, immunocytochemistry combined to image analysis of nuclear abnormalities) of PBMC nuclear, cytosolic and mitochondrial targets of antiretroviral drugs : A and B lamins, NF-B + I-B and proteasome activity assay, CD36 (glycosylation), mitochondrial Hsp70, ROS mitochondrial production, mitochondrial inner membrane potential, cytochrome C oxidase subunits 2 and 4 [Cell Biology, Timone CHU, Marseille] ; v/ genotyping the antiretroviral targets : lamin A (ZMPSTE24) and B (Rce1) processing proteases, Golgi SREBP-releasing proteases (MBTPS1 and S2), mitochondrial deoxynucleoside transporters (SLC25A4 to A6), mitochondrial proteases (MPPA, paraplegin) involved in processing of nuclear encoded proteins during their mitochondrial import ; quantitative PCR measurement of telomere length [Molecular Genetics, Timone CHU, Marseille]. Marseille's CIC-UPCET collaborated to the protocol design, will recruit control subjects and will be responsible for statistical treatment of data.

Eligibility

Ages Eligible for Study:	18 Years to 65 Years
Genders Eligible for Study:	Both
Accepts Healthy Volunteers:	Yes
Sampling Method:	Non-Probability Sample

Study Population

50 HIV1-infected naive patients (A group), apparied to 50 age- and sex-matched seronegative control (C group) subjects and 100 HIV1-infected patients in first line of antiretroviral therapy for at least 12 months (B group)

Criteria

Inclusion Criteria:

Age ≥ 18 years and <65 years Able to give written consent Covered by French Social Security Non infected by HIV-2

- - A group HIV1-infected naive patients
- -B group infected patients in first line of antiretroviral therapy for at least 12 months
- -C group HIV seronegative Confirmed by a fast test of screening of the HIV at day one of study

Exclusion Criteria:

- Age < 18 years and > 65 years
- Not Able to give written consent
- Not Covered by French Social Security
- Infected by HIV-2
- treated by statin or biphosphonat amino
- concomitant treatment: diabetic or testosteron

Contacts and Locations

Please refer to this study by its ClinicalTrials.gov identifier: NCT01038999

Locations

France

ANRS center from Marseille, Timone and Montpellier and Nice
country of French, France

Sponsors and Collaborators

French National Agency for Research on AIDS and Viral Hepatitis

Investigators

Principal Investigator:	Isabelle POIZOT-MARTIN	CHU Sainte Marguerite -Marseille
Principal Investigator:	Marie-Pierre DROGOUL	CHU Sainte Marguerite -Marseille
Principal Investigator:	Olivia FAUCHER	CHU Sainte Marguerite -Marseille
Principal Investigator:	Amélie MENARD	CHU Sainte Marguerite -Marseille
Principal Investigator:	Joëlle MICALLEF-ROLL	CHU Timone
Principal Investigator:	Jacques REYNES	CISIH CHRU Gui de Chauliac- Montpellier
Principal Investigator:	Pierre DELLAMONICA	CISIH CHU Nice
Principal Investigator:	Pierre CAU	INSERM UMR S910 MARSEILLE
Principal Investigator:	Catherine TAMALET	Laboratoire Virologie Marseille
Principal Investigator:	Bruno LACARELLE	Unité INSERM U911 Marseille
Principal Investigator:	Nicolas LEVY	Laboratoire Génétique Moléculaire Marseille
Principal Investigator:	Patrick ROLL	Laboratoire biologie cellulaire Marseille

More Information

Additional Information:

Related Info EXIT

No publications provided

Responsible Party:	Lucie Marchand/Project manager, French National Agency for Research on AIDS and Viral Hepatitis
ClinicalTrials.gov Identifier:	NCT01038999 History of Changes
Other Study ID Numbers:	2008-A00905-50
Study First Received:	December 23, 2009
Last Updated:	September 12, 2011
Health Authority:	France: Afssaps - French Health Products Safety Agency

Keywords provided by French National Agency for Research on AIDS and Viral Hepatitis:
Complementary Therapies

Additional relevant MeSH terms:

- HIV Infections
- Acquired Immunodeficiency Syndrome
- Lentivirus Infections
- Retroviridae Infections
- RNA Virus Infections
- Virus Diseases
- Sexually Transmitted Diseases, Viral
- Sexually Transmitted Diseases
- Immunologic Deficiency Syndromes
- Immune System Diseases
- Slow Virus Diseases

ClinicalTrials.gov processed this record on March 05, 2012

Contact Help Desk
Lister Hill National Center for Biomedical Communications, U.S. National Library of Medicine,
U.S. National Institutes of Health, U.S. Department of Health & Human Services,
USA.gov, Copyright, Privacy, Accessibility, Freedom of Information Act
