# Supplementary material for: HIV-1 Infection and First Line ART Induced Differential Responses in Mitochondria from Blood Lymphocytes and Monocytes: The ANRS EP45 “Aging” Study
Source: PLoS One. 2012 Jul 19;7(7):e41129. doi: 10.1371/journal.pone.0041129 (PMC3400613; doi:10.1371/journal.pone.0041129)
Supplement: Methods S1 — Cell viability and western blot procedures. (DOC) [file pone.0041129.s009.doc]

**Supporting information**

Perrin et al.: HIV-1 Infection and First Line ART Induced Differential Responses in Mitochondria from Blood Lymphocytes and Monocytes: the ANRS EP45 “Aging” Study.

**Supporting methods S1**

**Cell viability**

To standardize sample processing despite shipping delays between HIV Clinical Units (Montpellier, Nice and Marseille) and the Cell Biology Laboratory (Marseille), blood collected in EDTA Vacutainer tubes (Becton Dickinson) was rotated overnight before PBMC Ficoll isolation. Aliquots of 250,000 PBMCs were then incubated at 37°C, 5% CO_2_ for 3 h in RPMI medium supplemented with 10% foetal bovine serum and 2 mM L-glutamine before functional or morphological mitochondria analysis.

Cell viability was checked in a small number of HIV-1 infected patients at 4 different time points: blood sample reception; overnight rotation; PBMC isolation; 3 h incubation step. The percentage of dead cells was evaluated using 7-amino-actinomycin D probe (7-AAD, Beckman Coulter Inc). Blood samples or PBMCs were incubated with probes according to the manufacturer’s instruction for 20 minutes at RT. Blood samples were additionally incubated with CD45-FITC (Beckman Coulter Inc). Red blood cells were hypotonically lysed (100 µM EDTA, 150 mM NH_4_Cl, 1 mM KHCO_3_, pH 7.4). Fluorescence intensity was measured by flow cytometry (Navios, Beckman Coulter Inc) on an average of 20,000 PBMCs identified according to side scatter (SSC) and CD45 staining for blood samples, or forward and side scatter only for PBMCs. All data were analysed using FlowJo® software (Tree Star Inc).

The percentages of early apoptotic PBMCs were evaluated using annexin V-FITC/7-AAD kit (Beckman Coulter). Experiments were performed after PBMC ficoll isolation and after the 3 h incubation step. PBMC co-staining was performed according to the manufacturer’s instructions. Cells incubated overnight with staurosporin (1 μM; Sigma) were used as a positive control for apoptosis. Fluorescence intensity was measured by flow cytometry (Navios, Beckman Coulter Inc) on an average of 3,500 CD14+ monocytes and 10,000 lymphocytes identified according to forward/side scatter (FSC, SSC) (Figure S1).

**Western blotting of mitochondrial proteins**

Immediately after PBMC isolation, residual red blood cells, if any, were hypotonically lysed (100 µM EDTA, 150 mM NH_4_Cl, 1 mM KHCO_3_, pH 7.4). Cells were stored at -80°C.

Total PBMC proteins from 160 cohort participants were extracted by incubation at 4°C for 20 minutes in 100 μL of extraction buffer (20 mM HEPES (pH 7.5), 150 mM NaCl, 1 mM EDTA, 1% NP40 (v/v), 0.5% sodium deoxycholate, 0.1% SDS, 10 μg/ml aprotinin, protease inhibitor cocktail (complete EDTA-free, Roche, Mannheim, Germany), 0.5 mM PMSF, 1 mM DTT), and then centrifuged at 10,000 g for 10 minutes. Total proteins from human alveolar macrophages from non-HIV control subjects were used as a positive control. Protein concentration was determined using the BCA™ Protein Assay (Thermo Scientific).

20 µg of each lysate were loaded onto 10% Bis-Tris Criterion^TM^ XT precast gels (Biorad) and transferred on Immobilon-FL PVDF membrane (Millipore). Membranes were blocked for 1 hour in 1:2 diluted blocking buffer for near infrared fluorescent western blotting (Rockland). Primary antibodies against respiratory chain complex IV (Cytochrome c Oxidase, CoxIV) subunits 2 and 4 (CoxIV-2, 1:1000, Life Technologies; CoxIV-4; 0.5 µg/ml, Abcam) and VDAC-1/porin (1 µg/ml, Abcam) were incubated with the membranes overnight at 4°C. Incubation with glyceraldehyde 3-phosphate dehydrogenase (GAPDH) primary antibody (1:40,000, Millipore) for 1 h at RT was used as a total cell protein loading control. IR-Dye 700 conjugated anti-rabbit (1:5000, LI-COR Biosciences) and IR-Dye 800 conjugated anti-mouse (1:5000, LI-COR Biosciences) were incubated for one hour at RT. Bound antibodies were detected and analysed on an Odyssey® imaging V3.0 system (LI-COR Biosciences) according to the manufacturer’s instructions. Band intensities were measured and normalized to those of GAPDH (total cell protein loading control). Discriminant analysis using normalized protein ratios to GAPDH indicated that the overall western blotting procedure did not result in significant differences from one gel to another for all subjects belonging to the same group (not shown). The identification of ubiquitinylated CoxIV-4 was performed using antibodies against ubiquitinylated proteins (1:1000, Enzo Life Sciences) and CoxIV-4 (1 µg/ml, Abcam) for 1 h at RT. The identification of CoxIV-4 with uncleaved mitochondrial targeting signal was performed using 12% Bis-Tris Criterion^TM^ XT precast gels (Biorad) to enable better separation of proteins between 15 to 20 kDa.
